# Embryonic tissue differentiation is characterized by transitions in cell cycle dynamic-associated core promoter regulation

**DOI:** 10.1093/nar/gkaa563

**Published:** 2020-07-03

**Authors:** Joseph W Wragg, Leonie Roos, Dunja Vucenovic, Nevena Cvetesic, Boris Lenhard, Ferenc Müller

**Affiliations:** Institute of Cancer and Genomic Sciences, College of Medical and Dental Sciences, University of Birmingham, Birmingham, B15 2TT, UK; Institute of Clinical Sciences and MRC Clinical Sciences Centre, Faculty of Medicine, Imperial College London, London, W12 0NN, UK; Institute of Clinical Sciences and MRC Clinical Sciences Centre, Faculty of Medicine, Imperial College London, London, W12 0NN, UK; Institute of Clinical Sciences and MRC Clinical Sciences Centre, Faculty of Medicine, Imperial College London, London, W12 0NN, UK; Institute of Clinical Sciences and MRC Clinical Sciences Centre, Faculty of Medicine, Imperial College London, London, W12 0NN, UK; Institute of Cancer and Genomic Sciences, College of Medical and Dental Sciences, University of Birmingham, Birmingham, B15 2TT, UK

## Abstract

The core-promoter, a stretch of DNA surrounding the transcription start site (TSS), is a major integration-point for regulatory-signals controlling gene-transcription. Cellular differentiation is marked by divergence in transcriptional repertoire and cell-cycling behaviour between cells of different fates. The role promoter-associated gene-regulatory-networks play in development-associated transitions in cell-cycle-dynamics is poorly understood. This study demonstrates in a vertebrate embryo, how core-promoter variations define transcriptional output in cells transitioning from a proliferative to cell-lineage specifying phenotype. Assessment of cell proliferation across zebrafish embryo segmentation, using the FUCCI transgenic cell-cycle-phase marker, revealed a spatial and lineage-specific separation in cell-cycling behaviour. To investigate the role differential promoter usage plays in this process, cap-analysis-of-gene-expression (CAGE) was performed on cells segregated by cycling dynamics. This analysis revealed a dramatic increase in tissue-specific gene expression, concurrent with slowed cycling behaviour. We revealed a distinct sharpening in TSS utilization in genes upregulated in slowly cycling, differentiating tissues, associated with enhanced utilization of the TATA-box, in addition to Sp1 binding-sites. In contrast, genes upregulated in rapidly cycling cells carry broad distribution of TSS utilization, coupled with enrichment for the CCAAT-box. These promoter features appear to correspond to cell-cycle-dynamic rather than tissue/cell-lineage origin. Moreover, we observed genes with cell-cycle-dynamic-associated transitioning in TSS distribution and differential utilization of alternative promoters. These results demonstrate the regulatory role of core-promoters in cell-cycle-dependent transcription regulation, during embryo-development.

## INTRODUCTION

Understanding transcription regulation in development represents a challenge due to the dynamic nature of multiple cellular lineages continuously evolving into an increasingly complex organism. Increasing evidence suggest that cell cycle control affects transcription regulation in development (1–4), while the rules and regulatory ‘grammar’ on the level of cis-regulatory elements, associated with cell cycle regulation is little understood. A particularly difficult question is, how transcription is controlled in dynamically interacting embryonic cells of the embryo, which follow distinct cell proliferation fates independently from their lineage commitment?

Over the last decade, the core promoter, a stretch of DNA surrounding the transcription start site (TSS) of all genes, has emerged as a key site of transcriptional regulation, integrating signals received from multiple cis-regulatory elements (5,6). The advent of 5′ end transcript sequencing (e.g. Cap analysis of gene expression [CAGE]), has greatly enhanced our ability to interrogate the role the core promoter of a gene plays in transducing regulatory signals into gene transcription (7–10). Single base pair resolution analysis of TSS location (using CAGE) has revealed an immense diversity in the pattern of transcription initiation on the core promoter, from a narrow distribution of TSSs, with a single base pair dominant site (termed sharp promoters), to a dispersed pattern of TSS usage across the promoter (broad), with a spectrum of different promoter architectures between these two extremes (7–11). Investigation of divergent core promoter architectures have revealed these to be a strong indicator of distinct regulatory networks, acting on the core promoter, modulating cell behavior through transcriptional profile changes (10,12–14). This is of importance in understanding how key transitions in cellular behaviour, during embryonic development, are regulated at the level of transcription initiation. The role that promoter-associated gene regulatory networks play in development associated transitions in cell cycle dynamics (e.g. during differentiation) however, is poorly understood.

Embryonic development is marked by several dramatic transitions in the regulatory make up of cells, to permit changes and limitations in their potency, leading to the formation of an organized hierarchical body map. These transitions are often associated with changes in cell cycle dynamics, alongside shifts in transcriptional repertoire (1–4). This process commences with the fusion of two gamete cells into a single fertilized embryo. In many eukaryotes, including zebrafish, this is followed by a number of rapid, synchronous cell cycles, with embryonic behaviour exclusively controlled by maternally deposited factors. At the midblastula transition (MBT) the zygotic genome activates and this process is marked by a slowing of the cell cycle and a loss of synchrony (reviewed in (15,16)). We have previously shown that the transition in cell behaviour from the rapidly cycling synchronous divisions before MBT, to slower, asynchronous, divisions after MBT, accompanied by the activation of the zygotic genome, is marked by a switch in transcription initiation grammar from defined, W-box mediated transcriptional output, to a broader unrestricted initiation grammar, but confined by nucleosome positioning (17). Extensive regulatory reprograming is seen in other model organisms, during this period too, with the first stages of mouse embryo development marked by extensive chromatin remodelling, with lineage-specific expression of several chromatin modifiers, underscoring the potential role of gene regulatory networks in controlling cell fate decisions (18,19).

**Figure 1.**
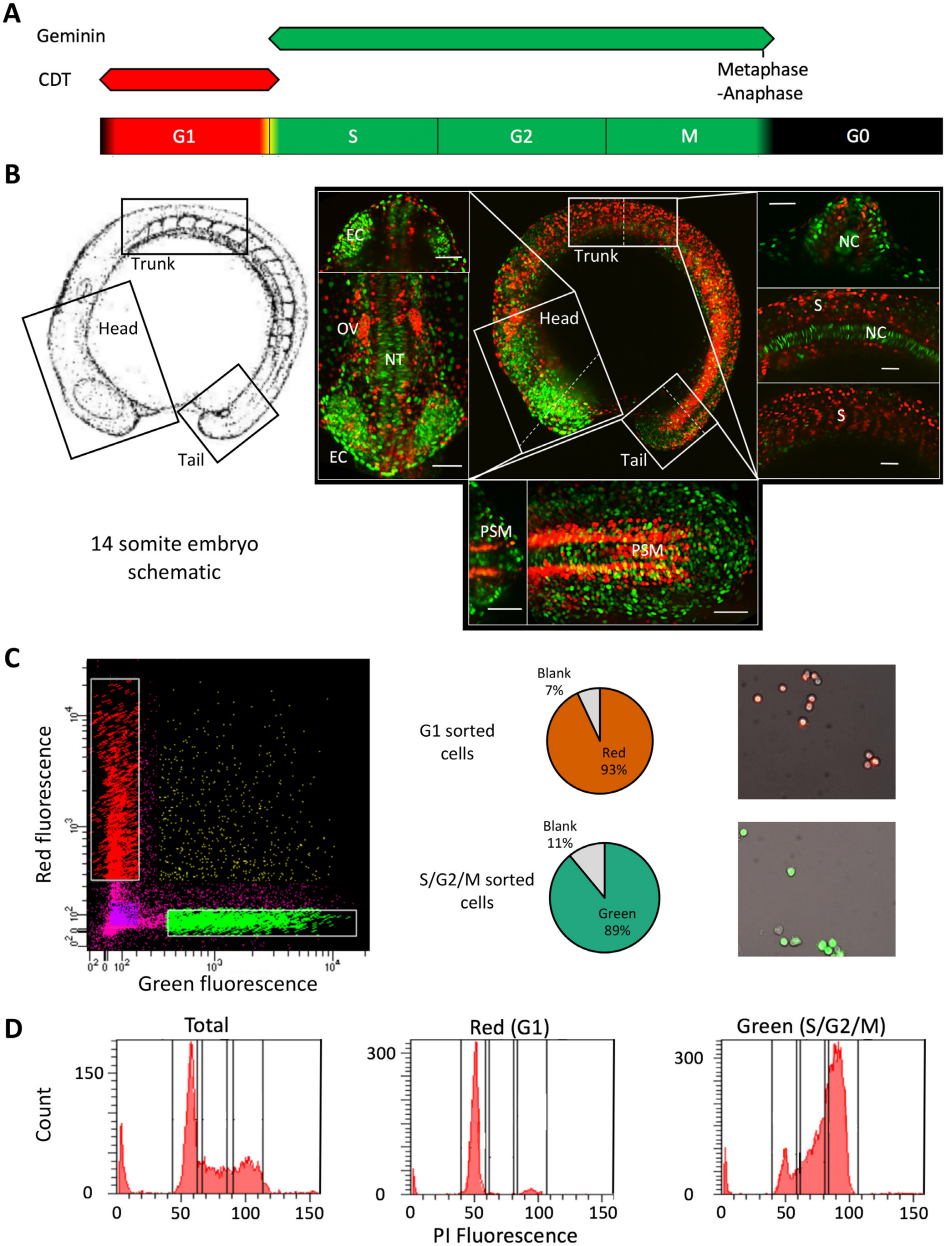
FACS mediated separation of cells by cell cycle stage in the developing embryo. (**A**) Schematic of fluorescence from the FUCCI system through the cell cycle with colours indicating phases marked by the fluorescent reporter genes. (**B**) Left panel; a schematic of the 14-somite embryos, reproduced with permission from (15) (© 1995 WILEY-LISS, INC.). Right panel; center: max projection fluorescent image of a 14-somite FUCCI transgenic embryo, showing the distribution of rapidly (green) and slowly cycling (red) cells. Surrounding panels: higher magnification views of the head, trunk and tail regions with alternate views shown in the top/right sections and cross-sections (denoted by the dashed lines) shown in the bottom/ left sections of each surrounding panel. Scale bar = 50 uM. Abbreviations, otic vesicle (OV), eye cup (EC), notochord (NC), somites (S), neural tube (NT), pre-somitic mesoderm (PSM). (**C**) FACS sorting of FUCCI embryos, from left to right, FACS plot showing gating, pie charts showing cell selection efficiency and representative fluorescent images of isolated cells. (**D**) Propidium Iodide DNA content analysis of isolated cells. FACS traces show a shift in isolated cell DNA content from a level in accordance with primarily Gap phase 1 cells (G1) (gated as P3) in the red population to principally S and G2 phase cells (gated in P4 and 5) in the green population, emanating from a mixed total population. Proportions are shown in Supplementary Table S1.

The Fluorescent Ubiquitination-based Cell Cycle Indicator (FUCCI) is a system that allows the visualization of cell cycle progression in living cells, through the fusion of fluorescent markers onto cell cycle phase restricted factors (often Cdt1 and Geminin) (20) as detailed in Figure 1A. Studies using this system in developing zebrafish have revealed that in subsequent stages of development, the process of cell differentiation is marked by a further slowing in cell cycle dynamics as tissue-lineages are specified (21). This is in agreement with *in vitro* studies of human and murine embryonic stem cells, showing that a key indicator of cell differentiation from pluripotency, are transitions in cell cycle dynamics from rapid cycling to a slower cycling identity, characterized by an elongated G1 phase (1–4). In addition to this, studies, investigating transitions from lineage defined multipotent stem cells to terminally differentiated cells, in both muscle (22) and liver development (5), have shown that this process is marked by wholesale depletion of RNA polymerase II regulatory cofactors. Additionally, various cell lineages on the path towards differentiation, appear to be regulated by distinct general transcription factors, forming preinitiation complexes (23–25). These findings suggest that the core promoter and associated basal transcription factors are an important yet unexplored component in the regulation of gene expression, controlling differentiation transitions.

We hypothesized that analysis of core promoter architecture during dynamic changes in cell cycle behaviour, in the developing embryo, will reveal the function of promoter regulation in key transitions of cellular behaviour during embryonic development. In this study, we aimed to explore, in an *in vivo* model of development, the role the core promoter plays in defining transcriptional output in cells undergoing differentiation coupled changes in cell cycle dynamics, through both promoter-level regulatory and behavioural changes. The zebrafish FUCCI transgenic system differentially marks cells in G1 and S/G2/M phases of the cell cycle and can therefore be used to separate rapidly and slowly cycling cells *in vivo*, by virtue of the cell cycle stage they primarily inhabit. This system was used to ask how cell cycle dynamics affect promoter usage during differentiation stages. We segregated cells as they go through differentiation coupled changes in cell cycle dynamics in segmentation stage embryos, which are characterized by marked spatial separation of differentiating cells with distinct cell cycle dynamics (21). CAGE 5′ end transcript analysis was used to interrogate the promoter features of these cell populations, to identify changes in the usage of transcriptional regulatory machinery. This investigation identified that the FUCCI system can successfully be used to segregate cells on the basis of cell cycle stage and cycling behaviour, as well as differentiation status, when this is marked by changes in cell cycle dynamics. Interrogation of promoter behaviour between populations revealed a distinct sharpening (condensation of TSS usage to a narrower region of the promoter) in promoter usage, in genes upregulated in slowly cycling, differentiating tissues. This event was associated with enhanced utilization of the TATA-box, in addition to Sp1 binding sites. A concurrent enhancement in CCAAT-box utilization in genes upregulated in rapidly cycling cells, was also observed, in genes with a broad range of TSS utilization in particular. A greater utilization of TATA-like and W-box motifs was also identified in rapidly cycling cells. A similar pattern of regulatory motif utilization was observed in genes transitioning in their TSS distribution, with differentially used alternative promoters and with temporal expression dynamics across the segmentation period. These changes were shown to be primarily associated with differential cell cycle dynamics, rather than tissue/lineage. This demonstrates a switch in core promoter associated transcriptional regulatory machinery utilization, leading to changes in promoter behaviour, as cells go through differentiation coupled changes in cell cycle dynamics. This investigation explores, for the first time, the regulation of tissue-lineage-specification on the promoter-level, in a whole organism, *in vivo* context.

## MATERIALS AND METHODS

### Zebrafish husbandry and embryo generation

All animal husbandry and associated procedures were approved by the British Home Office (Licence number: P51AB7F76). Zebrafish embryos were obtained by sibling crosses from adult FUCCI [(Tg(EF1α:mKO2-zCdt1(1/190))^rw0405b^/Tg(EF1α:mAG-zGem(1/100))^rw0410h^] (21) fish housed in the University of Birmingham fish facility. Zebrafish were bred and embryos raised and staged following standard protocols (15,26).

### Fluorescent imaging and image processing

Zebrafish embryos were decorionated using 1 mg/ml Protease from *Streptomyces griseus* (Sigma). For Lightsheet imaging (Zeiss), embryos were screened for fluorescence using a fluorescent microscope (Zeiss axio zoom V16), and mounted in a column of 1% low melting point agarose (Sigma). Zebrafish embryos were imaged using dual side illumination, with maximum intensity projection (MIP) processed images generated using the Zen Black imaging software (Zeiss).

For assessment of the relative proportions of red and green fluorescent cell populations, 10 embryos from each embryonic stage were imaged on a fluorescent microscope (Zeiss axio zoom V16) and pixel count for each fluorescent marker extracted using the ImageJ software package (27).

### Cell sorting and RNA preparation

Zebrafish were decorionated and selected as previously described at the 14-somite stage. Roughly 200 embryos were dissociated into a single cell suspension using enzyme-free cell dissociation buffer, PBS based (Gibco). Dissociated cells were pelleted and resuspended in Hanks balanced salt solution without calcium chloride or magnesium sulphate (Sigma), for sorting. Cells were fluorescence-associated cell sorted (FACS) into populations displaying red and green fluorescence. Validation of correct sorting was performed by propidium iodide DNA content analysis following manufacturer's conditions (Invitrogen). RNA was extracted from isolated cells using the miRNeasy kit (Qiagen). RNA quality was analysed by capillary electrophoresis (Bioanalyzer 2100, Agilent). All samples had an RNA integrity number (RIN) >9.

### CAGE library preparation and sequencing

NanoCAGE libraries were generated following a protocol described in (28). Pooled libraries, representing FACS sorted populations, in triplicate, were sequenced with the Illumina HiSeq 2500 50 cycles single-read run operation program, following manufacturer's protocol (Illumina).

### CAGE mapping and CTSS calling

The zebrafish genome assembly (Zv9) was downloaded from UCSC Genome Browser (29). Nano-CAGE reads were trimmed (15 bp from 5′end) to remove the linker and unique molecular identifier (UMI) region. For previously published data reanalysed in this study, raw CAGE sequencing data was downloaded from the repositories detailed in the respective publications (17,30,31). Reads were mapped using Bowtie (32), allowing a maximum of two mismatches and only uniquely mapping tags with MAPQ of 20. R/Bioconductor package CAGEr was used to remove the additional G nucleotide, due to the CAGE protocol, where it did not map to the genome (33). All unique 5′ ends of reads were defined as CAGE defined TSS’s (CTSS) and reads were counted at each CTSS per sample. These raw read counts were subsequently normalized based on a power-law distribution based on 10^6^ reads (34) and defined as normalized tags per million (tpm). After quality control per sample, a high level of inter-replicate correlation was observed and the biological replicates were merged for downstream analyses. One library of a biological replicate of 3 was excluded in the S/G2/M-phase merger, based on low complexity of the library.

### Calling transcriptional clusters

CTSS that were supported by at least 0.5 tpm in one of the samples were clustered based on a maximum allowed distance of 20 bp between two neighbouring CTSS. These transcriptional clusters (TCs) were then trimmed on the edges to obtain more robust boundaries of TCs, by obtaining the positions of the 10th and 90th percentiles of expression per TC. Only TCs with higher than 5 tpm expression were considered. Finally, TCs across all samples were aggregated if within 100 bp of each other to form consensus clusters (CC) for downstream analyses.

### Annotation

The CCs were annotated to the nearest reported TSS from Ensembl (danRer7) using the R/Bioconductor package ChIPseeker (35). We selected only CCs that mapped within 1 kb upstream of the reported TSS as well as CCs mapping to 5′ UTR.

### Differential gene expression analysis

The raw read counts were extracted for the CCs across triplicates described earlier and collapsed into total count per CC. DESeq2 R/Bioconductor package was used to define differential expression and the threshold of differential expression was set at adjusted *P*-value of <0.05. These results were cross referenced to the CC information of the merged samples. In cases of more than one CC mapping to the region, the CC with the highest expression was chosen to represent the region.

### Gene ontology

CCs were annotated with entrez gene IDs and analysed with GOstats R/Bioconductor package for overrepresentation of GO terms for biological processes. The up and down regulated genes were tested separately against all genes expressed amongst the samples.

### Tissue-specificity and cell cycle enrichment

The top 600 ranked human genes were downloaded from the Cyclebase database version 3.0. For each category of differential expression, the human orthologs (one to one & one to many) of zebrafish genes were determined using ‘mar2017.archive.ensembl.org’ archive of hg19. The peak time phenotype was determined by Cyclebase. Enrichment of cell cycle genes was determined with a permutation test (*n* = 10 000). Tau and entropy scores for tissue-specificity were determined by RNA-seq expression of eight adult zebrafish tissues from DanioCode series 391: brain, gill, heart, intestine, kidney, liver, muscle and spleen. Analysis of the relative representation of tissue-specific terms, was performed by cross-referencing differentially expressed genes with mRNA *in situ* expression data extracted from the ZFIN database, for wild-type zygote to 20 days post fertilization larval stages (https://zfin.org/downloads/wildtype-expression_fish.txt). This data was used to generate an anatomical specificity score that for each gene defines the pattern of spatial expression across development (Vucenovic and Lenhard, unpublished data). Data was then used to compare restrictedness of spatial localization of gene expression for two groups of genes, stratified across developmental time.

### Core promoter motif enrichment analysis

Position weight matrices (PWMs) for TATA-box, CCAAT-box, Sp1 binding site, INR, and YY1 binding site motifs were obtained from converting frequency matrices from JASPAR (7th release; 2018 (36)). Each CC was centred on the most expressed CTSS (the dominant TSS) and each sequence was scanned from 120 bp up and 50 bp downstream. A hit was reported if the scanned region contained a sequence with a 90% match to the PWM. For each group of differentially expressed genes, occurrence was counted and compared to the non-significant set of CCs per sample. Significance was assessed using Fishers' exact test. Obtained *P*-values were considered if <0.01. The log_2_ odds ratios were visualized as a heatmap. For W-box sequence analysis, all possible variations of a poly-W pentamer were identified and their relative frequency in the region 20–40 bp upstream of dominant TSS was measured. For each of the two groups, occurrences were counted and compared to the non-significant set of CCs per sample. Significance was assessed using Fishers' exact test.

### Promoter shape classification

Width of CCs (interquantile-width, IQW) were defined as the distance between the positions of the 10th and 90th percentiles of expression per CC. Sharp promoters were characterized by a width <10 bp, peaked broad promoters as ≥10 bp with one CTSS expressing more than 60% of expression of entire CC, and broad promoters as the remaining set.

### Alternative promoter utilization

The genomic location of 1612 twinned canonical and alternative promoters identified in (31), were extracted and intersected with the G1 and S/G2/M CAGE data generated for this paper. TPM values within these regions were calculated and the expression patterns of genes with a TPM >5 in either the canonical or alternative promoter region, in both the G1 and S/G2/M populations identified. Genes with a 2-fold change in the expression of the alternative promoter normalized to canonical promoter expression, were selected for further investigation (*n* = 79). These genes were segregated for the relative behaviours of alternative and canonical promoters as follows. *Canonical down* (expression of the canonical promoter in S/G2/M cells is >50% decreased vs. G1 cells [S/G2/M cano down] and Δ [difference in expression] S/G2/M vs. G1 for the alternative promoter is <25%), *Alternative down* (Δ cano <25%, Δ alt >50% [S/G2/M alt down]), *Canonical up* (Δ cano >50% [S/G2/M cano up], Δ alt <25%), Alternative up (Δ cano <25%, Δ alt >50% [S/G2/M alt up]). Situations falling outside of these criteria were discarded (*n* = 9).

## RESULTS

### Segregation of embryonic cells with distinct cell cycle dynamics

The FUCCI transgenic system, differentially marks cells in G1 and S/G2/M phases of the cell cycle (Figure 1A) and can therefore be used to separate rapidly and slowly cycling cells *in vivo*, by virtue of the cell cycle stage they primarily inhabit. Sugiyama *et al.* (21) demonstrated a switch in the ratio G1 versus S/G2/M marked cells in zebrafish undergoing somitogenesis, during the segmentation period, from primarily S/G2/M in early stages, to principally G1 in later stages, associated with cell differentiation. In order to further investigate this observation, longitudinal assessment of cell proliferation rate during zebrafish embryo development, using this system, was performed and revealed a spatial and tissue-specific separation in cell cycling behaviour across post-gastrulation embryos (Figure 1B, Supplementary Figure S1A, Movie S1). Cells, principally in the developing somites, had slowed cell cycling, displayed by an elongated period in G1-phase and therefore an accumulation of red fluorescent signal in the somites. In contrast, green (S/G2/M) cells marked primarily neuroectoderm derived lineages, such as optic cup, neural tube and notably, clearly identifiable cells in the notochord and circulating cells over the yolk ball (Figure 1B, Supplementary Figure S1A, Movie S1). 14-somite embryos showed the clearest spatial and tissue-specific segregation of cells on the basis of cell cycling dynamics (Figure 1B, Supplementary movie 2 and 3). In order to investigate the role of promoter associated transcriptional regulatory machinery in defining this transition, the 14-somite stage was selected for further investigation.

To this end, 14-somite FUCCI embryos were dissociated to a single cell suspension and segregated by fluorescence associated cell sorting (FACS) into cells in G1 (red) and S/G2/M (green) (Supplementary Figure S1B), with correct sorting confirmed by fluorescent imaging of the cells (Figure 1C). In order to validate that this process successfully segregates cells on the basis of cell cycle stage, segregated cells were subjected to DNA content analysis (Figure 1D). This analysis showed a marked enrichment for diploid (2N/G1) cells in the red, G1 segregated population, over the total background population (81 versus 39%) and enrichment for both 2-4N (S-phase) and tetraploid (4N/G2/M-phase) in the green, S/G2/M segregated population over total (27 versus 17% and 46 versus 22% respectively) (Figure 1D, Supplementary Table S1). This result confirms the successful segregation of cells on the basis of cell cycle stage using the FUCCI-transgenic system.

### Global transcription initiation patterns at known promoters

Next, we asked about the state of the mRNA transcriptome in fast and slow dividing cells of the embryo, with particular focus on the mRNA 5′ end, in order to identify features of promoter utilization. To achieve this goal, we chose a small cell number optimized protocol for detection of mRNA 5′ ends (nanoCAGE (28)), which reports steady state mRNAs quantitatively, and simultaneously informs about TSS usage and core promoter architecture (37). NanoCAGE was performed on three biological replicates of G1 and S/G2/M-phase segregated cells from the 14-somite stage zebrafish embryo, together with unsegregated cells (Total). CAGE reads were mapped to the zebrafish genome assembly (Zv9) and CTSSs assigned with a high level of inter-replicate correlation observed, with the exception of the S/G2/M (green) replicate 2, which was consequently excluded from further analysis (Supplementary Figure S2, Table S2). Based on this the biological replicates were merged for downstream analysis.

**Figure 2.**
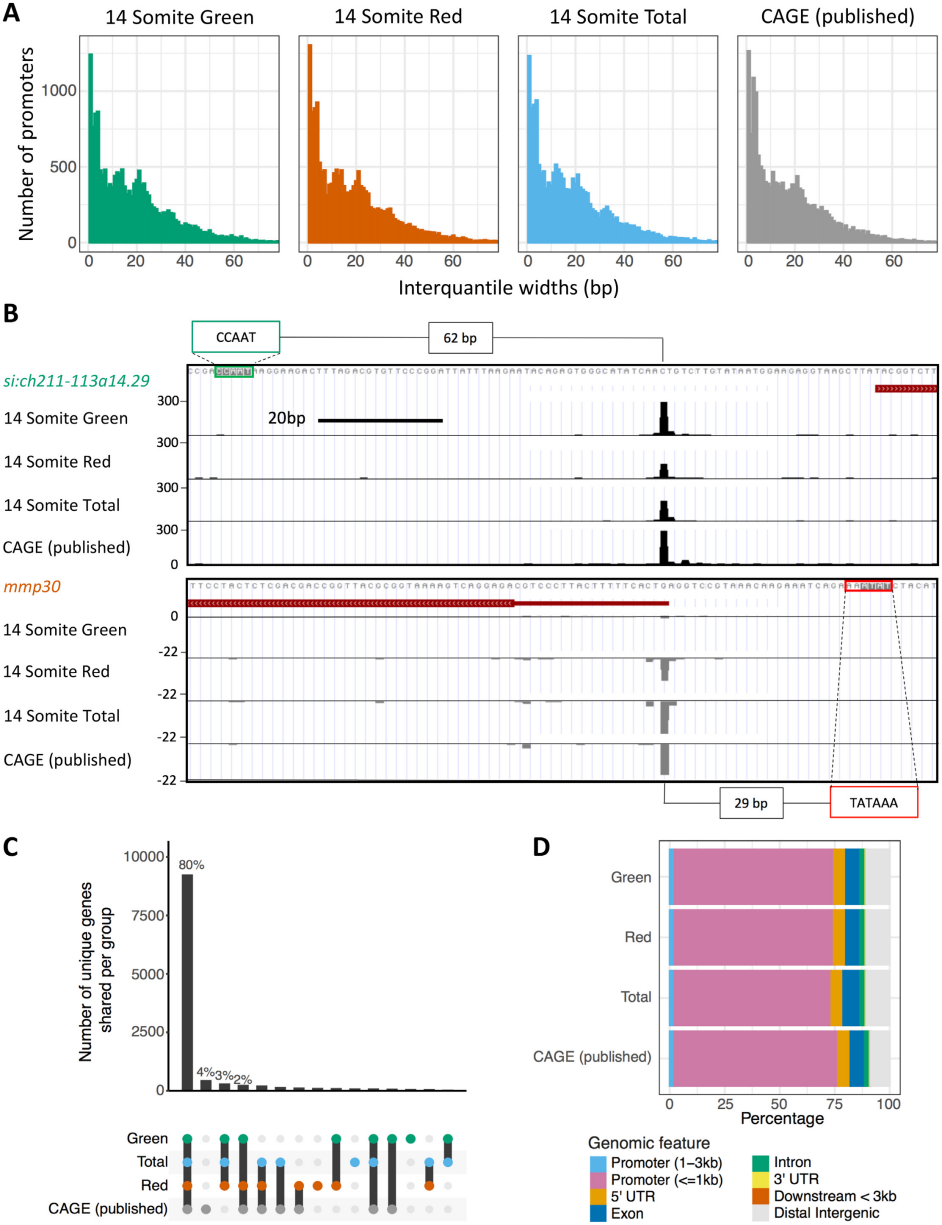
Overview of CAGE samples. (**A**) Histogram plots of the interquantile width of each Tag Cluster (TC). (**B**) UCSC genome browser view of *si:ch211-113a14.29* (an orthologue of human histone 2B) and *mmp30* (*matrix metallopeptidase 30*) showing consistent TSS distribution between nanoCAGE and published traditional CAGE. The position of putative regulatory motifs driving transcription are marked with distance from start of motif to dominant TSS position quantified. (**C**) A visualization of the overlap of TCs between the samples. The number of unique genes shared per group is shown in the lower half of the graph. (**D**) The number of TCs per sample mapping to genomic features. ‘Exon’, ‘5′ UTR’, ‘3′ UTR’ and ‘intron’ locations were extracted from the DanRer7 genomic build. ‘Promoter (≤1kb)’ = window 0–1 kb upstream of the gene start site, ‘Promoter (1–3 kb)’ = 1–3kb region upstream of gene start, ‘Downstream <3 kb’ = window 0–3 kb downstream of the gene end annotated in the DanRer7 genomic build and ‘Distal intergenic’ = all regions not covered in other classifications.

In order to validate that this approach successfully identifies the transcription start sites, the distribution of mapped CTSSs was compared with previously published CAGE data (generated using the tagging-CAGE version of the protocol (38)), from the 14-somite stage (31). Identified TSSs from each nanoCAGE sample, along with the tagging-CAGE, were grouped into consensus clusters (CCs) between samples and the distribution of TSSs (interquantile width [IQW]) within well expressed clusters (TPM ≥ 5) compared. This analysis revealed very similar interquantile width distributions between the nanoCAGE data and previously published data (31) (Figure 2A, Supplementary Figure S2B, Table S3). This is additionally exemplified by the very similar TSS distribution between samples of *si:ch211-113a14.29* (an orthologue of human histone 2B) and *mmp30* (matrix metallopeptidase 30) (Figure 2B). The determination of TSS distribution interquantile width is an established method for determining promoter shape, an important comparator of TSS utilization (7–10).

A high degree of overlap was additionally observed for cluster position between nanoCAGE and previously published data, with 80% the same genes represented in all samples (Figure 2C, Supplementary Tables S4&S5). Identified CTSSs were additionally mapped to genomic features (Figure 2D, Supplementary Figure S2C). The majority of CCs should fall within the core promoter region, <1 kb upstream of the annotated 5′ end of genes, as this is known to be the major site of transcriptional initiation and accordingly in this analysis ∼70% of TSSs mapped to this region (Supplementary Table S2), a similar level to previously published CAGE (Figure 2D). Taken together, these results indicate efficient isolation of gene promoter activities in cycling cells of differentiating embryos.

### Transcriptomes of G1 and S/G2/M cells reflect differential cell cycle and tissue-specific identities

**Figure 3.**
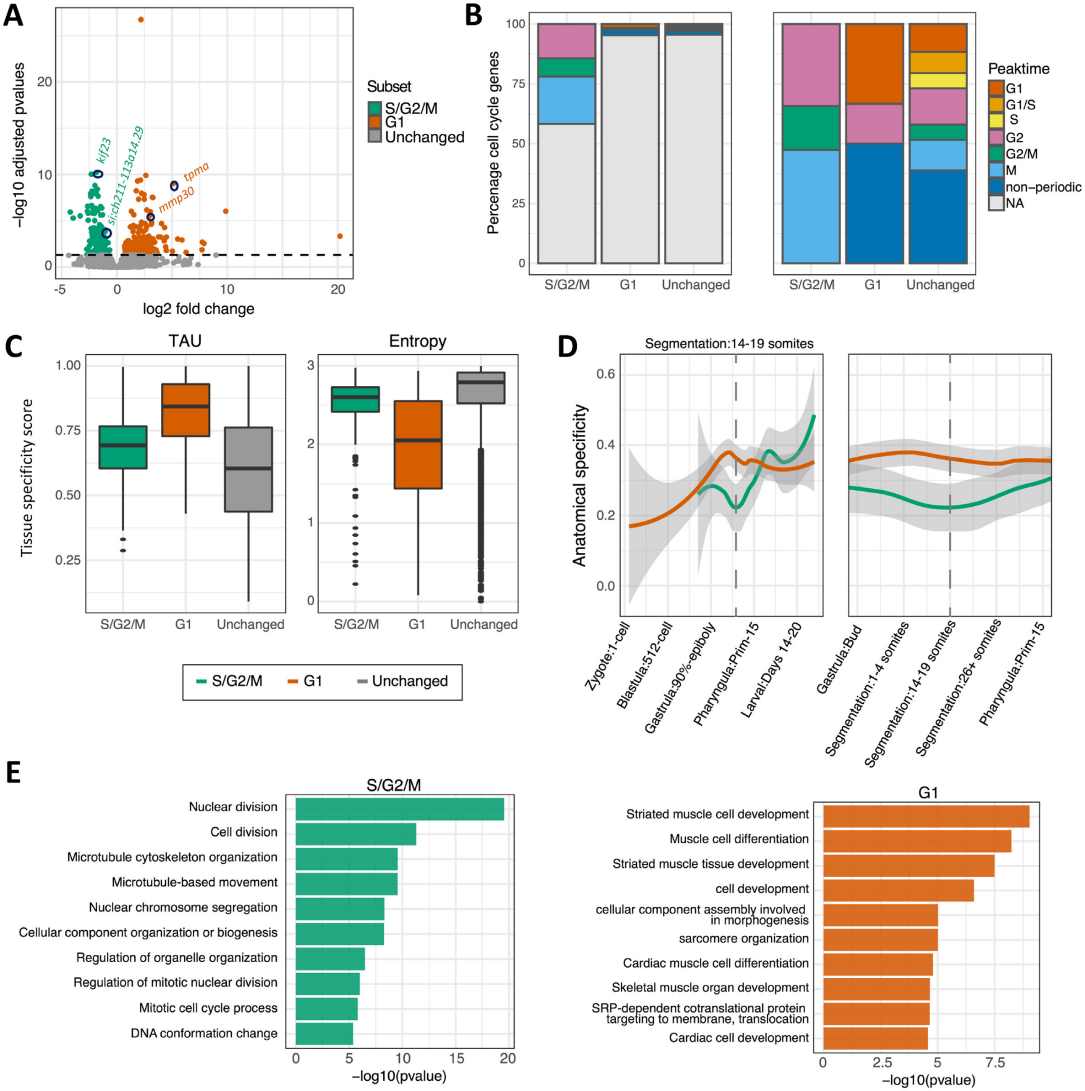
Classification of genes differentially expressed between G1 and S/G2/M segregated cells. (**A**) Volcano plot of all consensus clusters (CCs) in known promoter regions, coloured by significance. Location of genes shown in Figure 2B as well as the identity of top significant differentially expressed genes between populations highlighted by circles and gene names. (**B**) Bar plot of the percentage of promoters overlapping a human annotated cell cycle periodic gene from Cyclebase for each differentially expressed group and the CCs unchanged between the two groups. Left panel, full sample groups (*n* = 138, 190 and 8406, S/G2/M, G1 and unchanged respectively). Right panel, cell cycle periodic genes (*n* = 43, 6 and 246, S/G2/M, G1 and unchanged respectively). (**C**) Box plot of tissue specificity scores, based on adult zebrafish tissue for genes upregulated in G1 and S/G2/M and unchanged. (**D**) Anatomical specificity scores, based on expression in embryonic zebrafish tissues, for genes upregulated in G1 and S/G2/M. Left panel, anatomical specificity scores across development with key stages marked on x-axis. Right panel, anatomical specificity scores around stage at which the samples were collected (14-somite). Grey shading marks standard error, dashed line marks 14–19 somite stage. (**E**) Gene ontology of biological processes with their corresponding *P*-values.

CAGE is comparable to RNA-seq as a robust tool for quantitative transcriptomic analysis (31,37,39). Therefore, in order to profile the identity of cells segregated by cell cycle dynamics, and to identify differentially regulated genes, the expression of the promoter-associated consensus clusters was compared between samples. 12,865 consensus clusters were found to be shared between G1 (red) and S/G2/M (green), and clusters with a significant (P[adj]<0.05) change in expression between the populations identified (*n* = 190 [up regulated in G1], *n* = 138 [up regulated in S/G2/M]) (Figure 3A, Supplementary Table S6). This result indicates a large degree of overlap in the transcriptomes of the two populations, while differential regulation of a subset of transcripts opens the way to address their promoter regulation.

Fluorescence microscopy analysis of cell cycle dynamics in embryo development, revealed significant lineage-specific segregation of cells on the basis of their cell cycle dynamics (Figure 1B). Notably, the FUCCI system indicates highly dynamic variation in the distribution of cell populations segregating into fast and slow dividing groups and domains in the embryo (Supplementary movies 2 and 3). Therefore, differentially expressed genes in cells segregated by cell cycle stage, in this context, may represent both cell cycle and lineage-specification related differences. In order to determine the contribution of each of these elements to the populations of differentially expressed genes, they were cross-referenced with a databases of cell cycle periodic genes (CycleBase) (Figure 3B) and tissue-lineage-specific genes, as determined by expression patterns in both adult and embryonic tissues (Figure 3C and D respectively).

Analysis of differentially expressed genes revealed a significant enrichment for cell cycle periodically expressed genes upregulated in the S/G2/M (green) population (46%, *P* < 0.0001), with a peak of expression between G2 and M phase of the cell cycle (Figure 3B, Supplementary Table S6). Conversely only 3.2% of genes upregulated in the G1 (red) population were cell cycle periodic. A significant majority of these however had peak expression in G1. This is in contrast to the group of genes with unchanged expression, where the periodicity of genes is more evenly distributed between cell cycle stages (Figure 3B).

We hypothesized that differential expression in cells with distinct cell cycle dynamics is a result, at least in part, to distinct cell cycle behaviour of cell-lineages, which are distinct in nature, as well as represent varying levels of cell differentiation state. To test this, we asked about the contribution of lineage differentiation to selective expression of genes.

Analysis of the tissue-lineage-specificity of differentially expressed genes revealed a clear enrichment in those upregulated in the G1 (red) population over the other populations (demonstrated by a higher average Tau and lower average entropy score) (Figure 3C). Analysis of the anatomical specificity of G1 upregulated genes across embryo development, revealed this enrichment to be highly specific to the segmentation stage of development, from which the samples were collected (Figure 3D). These results suggest that a contributing factor, leading to differential expression between the populations, is cellular replication on the part of the S/G2/M (green) population and tissue-specification on the part of the G1 (red) population. This observation is supported by the gene ontology of the differentially expressed genes, revealing in the S/G2/M (green) population, a clear enrichment for genes involved in DNA and chromatin processing, key for rapidly cycling cells, and in the G1 (red) population, enrichment for muscle development associated gene expression (Figure 3E).

In order to further dissect the contribution of different cell types and developing lineages to the slowly versus rapidly cycling (G1 and S/G2/M) populations, differentially expressed gene sets were cross-referenced with the ZFIN database of gene with known tissue and spatial specific expression at the 14–19 somite stage (Supplementary Figure S3). The majority of genes did not match tissue-specific terms (72% [S/G2/M], 61% [G1] and 81% [Unchanged]) representing the fact that a minority of genes are tissue-specific and many tissues have not specified at the stage. This data does demonstrate however that the differentially expressed genes between the G1 and S/G2/M populations contain a disproportionate number of tissue-specific terms, particularly in the G1 population, in agreement with previous findings. Analysis of the relative contribution of genes with tissue-specific expression revealed a strikingly divergent expression pattern between populations (Supplementary Figure S3), with somite and muscle specific terms highly enriched in the G1 population, in agreement with gene ontology analysis and previously discussed fluorescence imaging (Figure 1B). Representation of terms related to the viscera, peripheral tissues (such as the periderm) and extra-embryonic tissues was also enriched in the G1 population, representing a population of cells starting to specify tissues within the embryo. In agreement with gene ontology analysis (Figure 3E), representation of proliferative and pluripotent cell types (such as the germ layers and proliferative region) was greatly enriched in the S/G2/M population. Interestingly, fluorescence imaging analysis (Figure 1B), suggest spatial distribution bias in the S/G2/M population compared to the G1 population, thereby S/G2/M phase green cells are particularly enriched in the eye cup and neural tissues. This apparent tissue bias is not borne out on the transcriptional level however, with neural and sensory tissue terms fairly evenly represented between populations (Supplementary Figure S3). A closer inspection of the tissue distribution of red and green cells however, suggest that neural lineages share both fast and slowly dividing cells (Figure 1B, surrounding panels). Global transcriptomic analysis will reveal most enriched tissues, but will not reflect tissue-specificity of cell cycle regulation. Nevertheless, this analysis shows that the dynamics of cell cycle regulation follows certain trends, which manifests as an enrichment for the transition to a tissue-specific expression profile, marked by cell cycle dynamic changes, as seen in the slowly cycling, G1 population.

### Genes differentially expressed between G1 and S/G2/M cells utilize different core-promoter classes and regulatory elements

**Figure 4.**
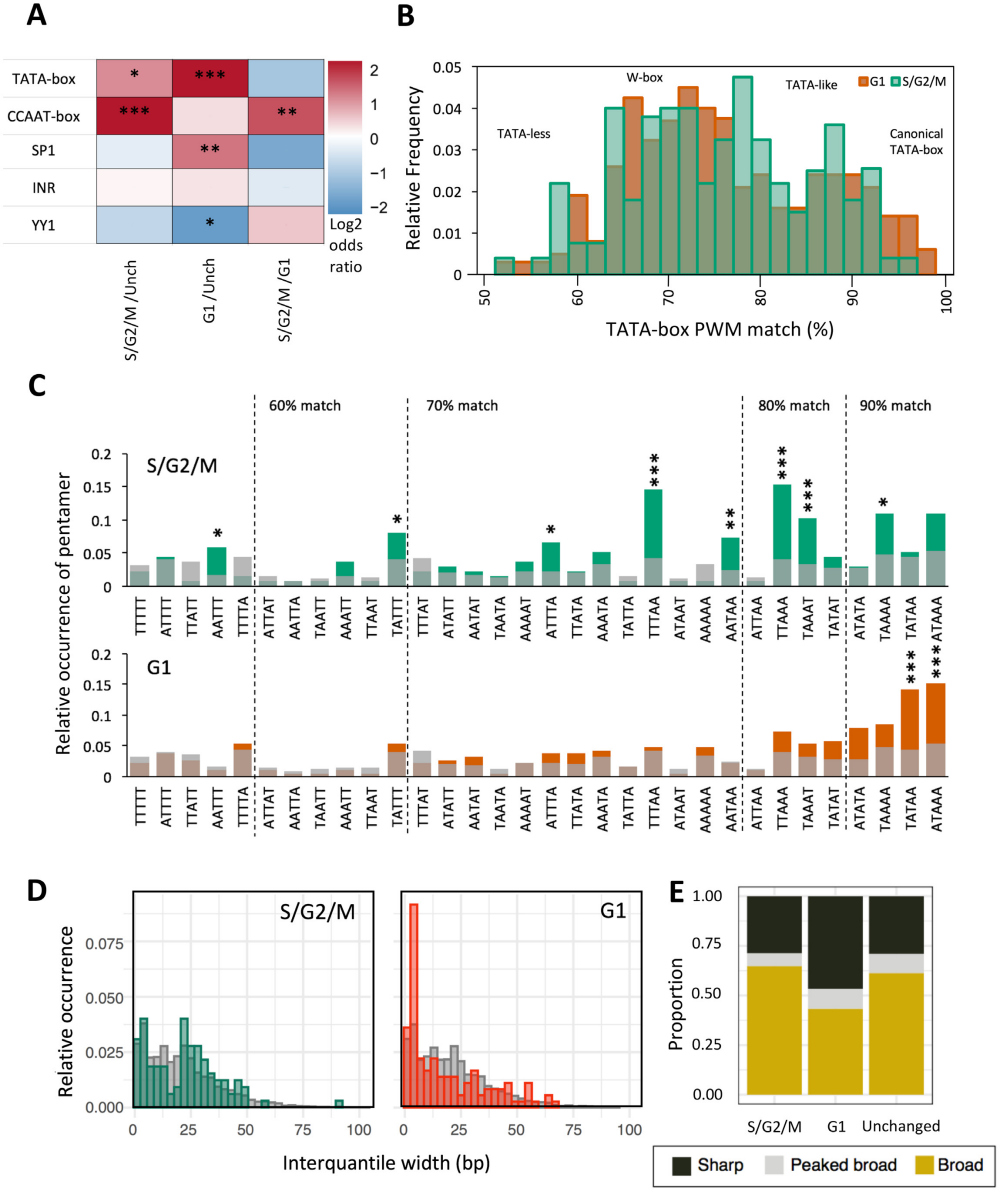
Core promoter architecture. (**A**) Heatmap visualizing the log_2_ odds ratio of occurrence of core promoter motifs between genes upregulated in G1 and S/G2/M and unchanged (*P< 0.05, **P< 0.01, ***P< 0.001, Fisher's exact test). (**B**) Distribution of position weight matrix (PWM) match (%) to TATA-box in the region –40 to –20 bp upstream of the dominant TSS in genes upregulated in S/G2/M and G1 populations. (**C**) A/T pentamer relative occurrence 20 to 40 bp upstream of TSS of genes upregulated in S/G2/M (green), G1 (red) and unchanged expression (grey). Ordering of pentamers is by best-fit match to the TATA PWM incremental thresholds shown. Pentamers significantly enriched in each group relative to pentamer occurrence in genes with unchanged expression (*P< 0.05, **P< 0.01, ***P< 0.001, Fisher's exact test). (**D**) Consensus cluster interquantile width in genes upregulated in S/G2/M (green) and G1 (red) and with unchanged expression (grey) visualized as a histogram. (**E**) Promoter shape distribution per group. Classifications; sharp (at least 90% of the expression from the promoter emanating from TSSs within 10bp of one another (IQW < 10) and ≥60% of expression emanating from a single dominant TSS); Sharp with broad background (≥60% of expression emanating from a single dominant TSS, but IQW ≥ 10) and Broad (<60% of expression emanating from a single dominant TSS and IQW ≥ 10).

As revealed by the analyses detailed above, a clear transition in cell population identities is occurring during the segmentation period in these embryos, marked by a dramatic phenotypic change (speed of cell cycle) and a divergence in transcriptional output. In order to determine whether differential gene expression, associated with cell cycle dynamics, is also associated with changes in core promoter regulatory element distribution, the frequency of known, regulatory motifs 120 bp upstream and 50 bp downstream of the dominant TSSs of each promoter was determined. Both groups of differentially expressed genes showed marked changes in motif utilization compared to genes with unchanged expression (Figure 4A, Supplementary Table S7). In the S/G2/M population there was a statistically significant enrichment for the NF-Y factor associated CCAAT-box, upstream of the TSS (*P*-value = 4.22 × 10^−12^). In the G1 population there was also a strong enrichment for a canonical TATA-box (*P*-value = 2.04 × 10^−7^), enrichment for Sp1 binding sites (*P*-value = 0.002), and a depletion for a YY1 binding site motif (*P*-value = 0.002). Strikingly, CCAAT-box and TATA-box relative strength of enrichment were inverse for S/G2/M and G1 enriched genes, relative to those with unchanged expression (Figure 4A). Motif enrichment in each case was also specific, being positionally restrained relative to the TSS (Supplementary Figure S4A). Examples of differentially expressed gene promoters, containing CCAAT/TATA-box motifs, are shown in (Figure 2B).

Besides altered utilization of specific regulatory motifs, previous analyses of development linked changes in promoter utilization, such as during zygotic genome activation (17), have found it to be associated with changes in the utilization of the W-box, an A/T (WW) rich stretch with similar positional constraint as the TATA-box to the TSS. In order to investigate whether this motif is differentially used, the relative frequency of WW-dinucleotides and TATA-box (>90% PWM match), 120 bp upstream and 50 bp downstream of the dominant site of transcription, in differentially expressed genes was calculated (Supplementary Figure S5). TATA-box frequency analysis was done alongside to differentiate TATA and W-box frequency, as a canonical TATA-box will appear as a WW dinucleotide enrichment in this analysis. This analysis confirmed TATA-box enrichment in genes upregulated in G1 (∼30 bp upstream of the TSS), but no enrichment in WW dinucleotide frequency between the G1 and S/G2/M differentially expressed gene sets (Supplementary Figure S5). This suggests that TATA and A/T rich motifs (such as W-box and TATA-like) may be differentially used between G1 and S/G2/M populations. W-box and TATA-like motifs are distinct from the canonical TATA-box by virtue of a looser motif specificity (17,40). In order to investigate their relative utilization, the approximate location of the TATA-box (20–40bp upstream of the TSS) was analysed for TATA-box position weight matrix (PWM) match (Figure 4B, Supplementary Figure S4B). This analysis revealed a shift from canonical TATA (>90% match) utilization enriched in G1, to 75–90% match (previously identified as distinct TATA-like and W-box motifs (17,40)) enriched in S/G2/M. W-box motifs, ∼5bp stretches of A/T bases, ∼30 bp upstream of the TSS (17,40), constitute a highly diverse population in terms of sequence identity. To investigate whether specific forms of W-box are enriched in each population, analysis of the relative occurrence of poly-W pentamers, 20–40 bp upstream of the TSS was performed. This analysis revealed that four TATA-divergent poly-W pentamers (TTAAA, TAAAT, TTTAA, AATAA) were significantly enriched (*P* < 0.001–0.01) in the promoters of genes upregulated in S/G2/M cells (Figure 4C, Supplementary Table S8). Two pentamers (TATAA, ATAAA) were significantly enriched (*P* < 0.001) in gene promoters upregulated in G1, however in 90% [21/23] (TATAA) to 100% [21/21] (ATAAA) of these cases the sequence formed part of a canonical TATA-box rather than a W-box. Overall this suggests a change in the degree of utilization of canonical vs. non-canonical TATA regulatory elements in genes differentially expressed between G1 and S/G2/M populations.

Canonical TATA-box utilization is strongly associated with sharp promoters, where a single or condensed cluster of TSSs are used in the promoter (7–9) and is associated with high level of expression often associated with structural genes (10). In order to determine whether enhanced TATA utilization is associated with a difference in the shape of promoter utilization, the distribution of TSSs (interquantile width [IQW]) within clusters was determined and compared between samples (Figure 4D). Additionally, consensus clusters were classified into classes on the basis of the pattern of TSS utilization (Figure 4E). Classifications were as follows; sharp (at least 90% of the expression from the promoter emanating from TSSs within 10 bp of one another (IQW<10) and ≥60% of expression emanating from a single dominant TSS); Peaked broad (≥60% of expression emanating from a single dominant TSS, but IQW ≥ 10) and Broad (<60% of expression emanating from a single dominant TSS and IQW≥10) (7). This analysis identified a distinct enrichment in sharp promoter utilization in G1 versus S/G2/M and non-significantly differentially expressed genes (43.7% versus 29.0% and 29.2% respectively) (Figure 4E). In order to determine whether this relative sharpening of promoter utilization in G1 differentially expressed genes was due to increased TATA utilization, relative TATA motif frequency (>90% PWM match) was determined in the promoter proximal region (120 bp upstream and 50 bp downstream of the dominant site of transcription) in G1 versus S/G2/M differentially expressed genes, as previously described, but segregated by promoter shape (sharp, peaked broad and broad) (Supplementary Figure S5). This analysis showed that indeed TATA-box utilization is highly enriched in sharp promoters over other behaviours, interestingly however this is only true in the gene set upregulated in G1. In the S/G2/M upregulated gene set promoter shape was only weakly associated with TATA utilization (Supplementary Figure S5). WW-dinucleotide frequency analysis done in an identical manner also showed no association with promoter shape. Collectively these findings suggest that enhanced TATA utilization and a greater proportion of sharp promoters are associated in genes upregulated in slowly cycling G1 cells (Figure 4D and E, Supplementary Figure S5).

### Differential core-promoter class and regulatory element utilization between S/G2/M and G1 populations is tissue-independent

As illustrated in Figure 3 and Supplementary Figure S3, differential tissue representation contributes alongside cell cycle dynamics in defining differential expression of promoters in the G1 and S/G2/M populations. In order to dissect whether the reported changes in promoter motif utilization and TSS distribution are linked to differential cell cycle dynamics, or is rather the product of distinct tissue identities, promoter behaviour analysis was repeated on tissue-specific genes alone, non-tissue specific genes, and a tissue independent gene set (Supplementary Figure S6A (i), (ii) and (iii) respectively). Tissue and non-tissue specific gene sets were segregated as described in Supplementary Figure S3 (tissue-specific: *n* = 38 [S/G2/M], 74 [G1], 1577 [unchanged]; non-tissue specific: *n* = 100 [S/G2/M], 116 [G1], 6849 [unchanged]. For the tissue independent gene set, promoters assigned to tissues represented in both populations (e.g. neural tissues and sensory tissues), or with no tissue specificity, were selected (*n* = 120 [S/G2/M], 141 [G1], 7633 [unchanged]). Analysis of motif frequency in the promoter region, 120 bp upstream and 50 bp downstream of the dominant site of transcription, revealed a consistent, significant enrichment for CCAAT-box utilization in the S/G2/M population of each gene set, albeit smaller in the tissue specific population (*P* = 0.005–0.0001, Fisher's exact test). This analysis also revealed a highly significant enrichment for TATA-box utilization in the G1 population of each gene set (*P* < 0.0001, Fisher's exact test), albeit larger in the tissue specific population, alongside an enrichment in Sp1 binding site utilization in the tissue independent gene sets (Supplementary Figure S6A and B).

This represents a similar pattern of motif utilization to the complete gene set (Figure 4A versus Supplementary Figure S6B). Analysis of promoter interquantile width (IQW) distribution in the tissue independent gene set (iii), showed a distinct narrowing and broadening of TSS distribution in G1 and S/G2/M enriched genes respectively, relative to genes with unchanged expression (Supplementary Figure S6B). The G1 sharpening however was less pronounced than in the analysis on the complete gene set (Figure 4D versus Supplementary Figure S6B). The tissue specific gene set was too small to accurately perform IQW analysis. Overall these analyses revealed that the reported pattern of promoter motif utilization and TSS distribution in S/G2/M versus G1 cells is characteristic of these populations, independent of the tissue specificity of the gene sets. However the differential tissue component between populations does somewhat contribute to G1 TATA enrichment and narrow TSS distribution.

### Gene promoters with marked change in TSS distribution between populations display differential regulatory element utilization

**Figure 5.**
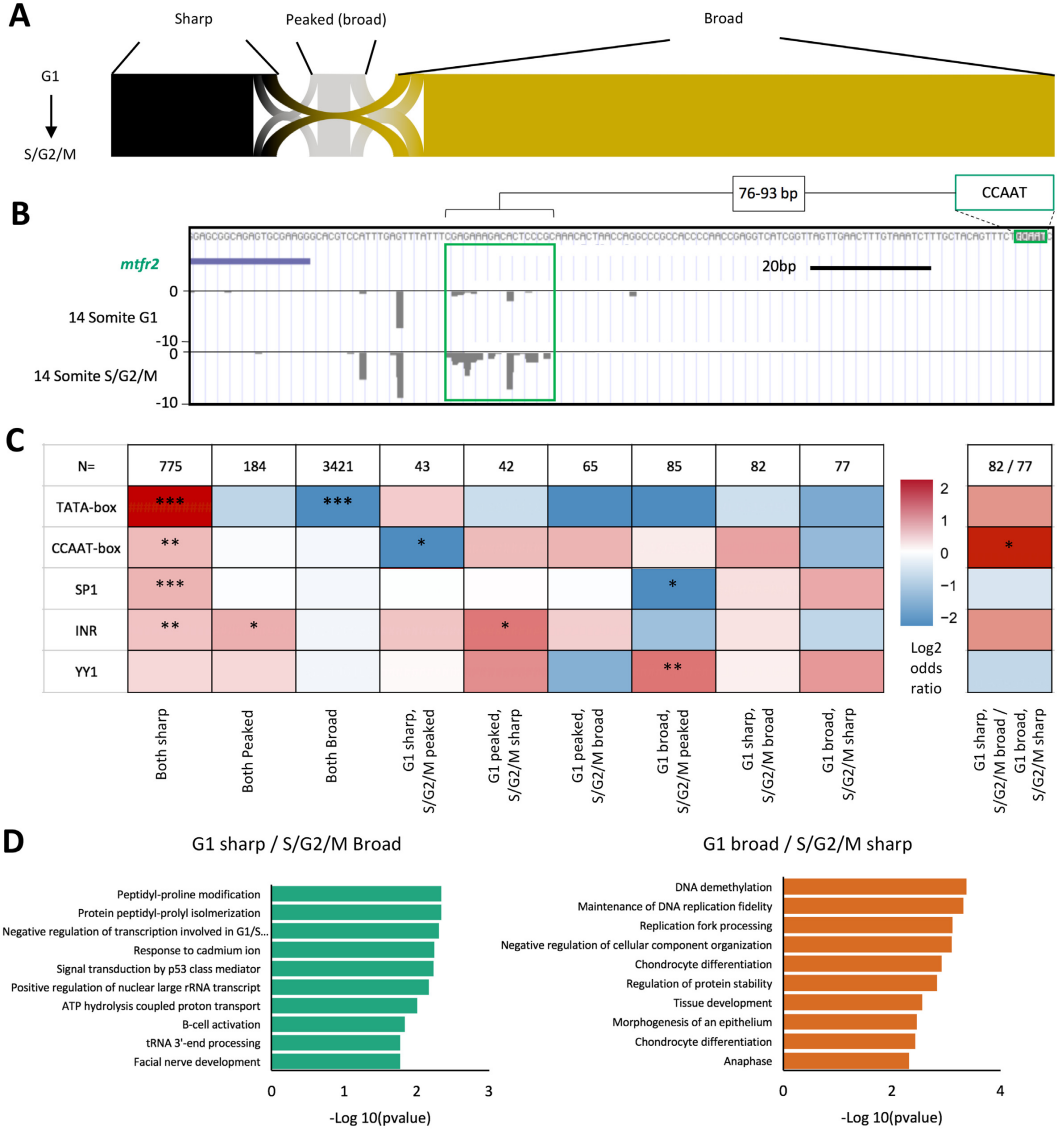
Core promoter shape transition. Promoter interquantile width (IQW) was measured and dominant TSSs assigned for consensus clusters (CCs) with at least 10 tpm expression in both the G1 and S/G2/M populations (*n* = 4774). Promoters were segregated into three groups based on promoter TSS distribution (shape), sharp (IQW < 10 bp), peaked broad (IQW > 10 bp, dominant TSS > 60% of CC expression) and broad (IQW > 10 bp, dominant TSS <60% of CC expression). (**A**) Sankey plot of promoter shape correspondence between G1 and S/G2/M. (**B**) UCSC genome browser view of the *mitochondrial fission regulator 2* (*mtfr2*) promoter with a shape change transition between populations, broadening TSS distribution in the S/G2/M population versus G1. A proximal regulatory element (CCAAT-box) is highlighted along with its spatial proximity to the main region of differential TSS utilization in this promoter (green box). (**C**) Heatmap visualizing the log_2_ odds ratio of selected promoter motif occurrence for promoters transitioning in shape. Left heat map, occurrence is scored in each group versus the rest of the dataset of shared CCs >10 tpm expression (*n* = 4774). Right heatmap, comparison is as shown (*P< 0.05, **P< 0.01, ***P< 0.001, Fisher's exact test). (**D**) Gene ontology of biological processes of genes with promoters transitioning from sharp to broad between populations, with corresponding *P*-values shown.

Analysis of genes differentially expressed between the G1 and S/G2/M population (Figures 3 and 4) show distinct differences in promoter behaviour (sharper promoter utilization in slowly cycling G1 cells) and the regulatory networks associated with their expression (often involving the differential usage of TATA or CCAAT-boxes). As described in Figure 2A, the global distribution of promoter usage is unchanged between populations, however analysis of TSS distribution, in promoters highly expressed (TPM > 10) in both populations, revealed a significant proportion of genes transitioning in promoter shape (sharp, peaked broad and broad) between populations (394/4774, 8.3%) (Figure 5A). This event is potentially representative of a transition in dominant promoter regulatory network (7–9). *Mitochondrial fission regulator 2* (*mtfr2*) for example, a gene associated with cell proliferation in human (41), displays a shape change transition between populations, broadening TSS distribution in the S/G2/M population versus G1. This gene additionally contains a CCAAT-box within its promoter, proximal to the shape change event (Figure 5B).

This population of promoters show context dependent changes in their utilization. They therefore could represent an important population defining alterations in the promoterome (and therefore the transcriptome) of cells undergoing differentiation coupled changes in cell cycle dynamics. In order to determine whether, like differentially expressed promoters, these transitioning promoters are marked by different regulatory machinery to each other, promoter proximal regulatory motif analysis, was performed, as before (Figure 5C). This analysis revealed, in line with previous studies (10,12–14), significant enrichment for the TATA-box motif in genes that remained sharp in both populations and a depletion of TATA in genes where broad promoter utilization is retained (Figure 5C). Interestingly CCAAT-box and Sp1 binding site motif frequency were also enriched in the population of genes with shared sharp initiation (though to a 17-fold lesser degree than the TATA-box), despite both being associated with broad promoters in vertebrates (6,11,42). Of note YY1 binding-site motif occurrence is enriched in genes with a condensed TSS distribution in the S/G2/M population relative to G1 (G1 peaked/S/G2/M sharp, G1 broad/S/G2/M sharp, G1 broad/S/G2/M peaked), the latter significantly (n<0.01). Of greatest interest however are gene promoters where there is a significant shift in TSS distribution (from sharp to broad) between populations. Direct comparison of these populations (G1 sharp/S/G2/M broad versus G1 broad/S/G2/M sharp) revealed a significant (*P* < 0.05) enrichment for CCAAT-box occurrence in the former population (Figure 5C), further supporting the association of the CCAAT-box motif with differential promoter usage between populations. Gene ontology analysis of these broad-to-sharp shifting populations revealed a mixed set of terms with both proliferation and differentiation genes represented (Figure 5D). This suggests that transitions in the TSS distribution on the promoter has a subtler effect on transcriptional output, than on differentially expressed genes, however both processes are driven by the utilization of similar regulatory motifs and argue for distinct activity of general transcription factor complexes on promoters, in fast and slow dividing cells.

### Differential utilization of alternative promoters between cell cycle dynamic divergent populations

**Figure 6.**
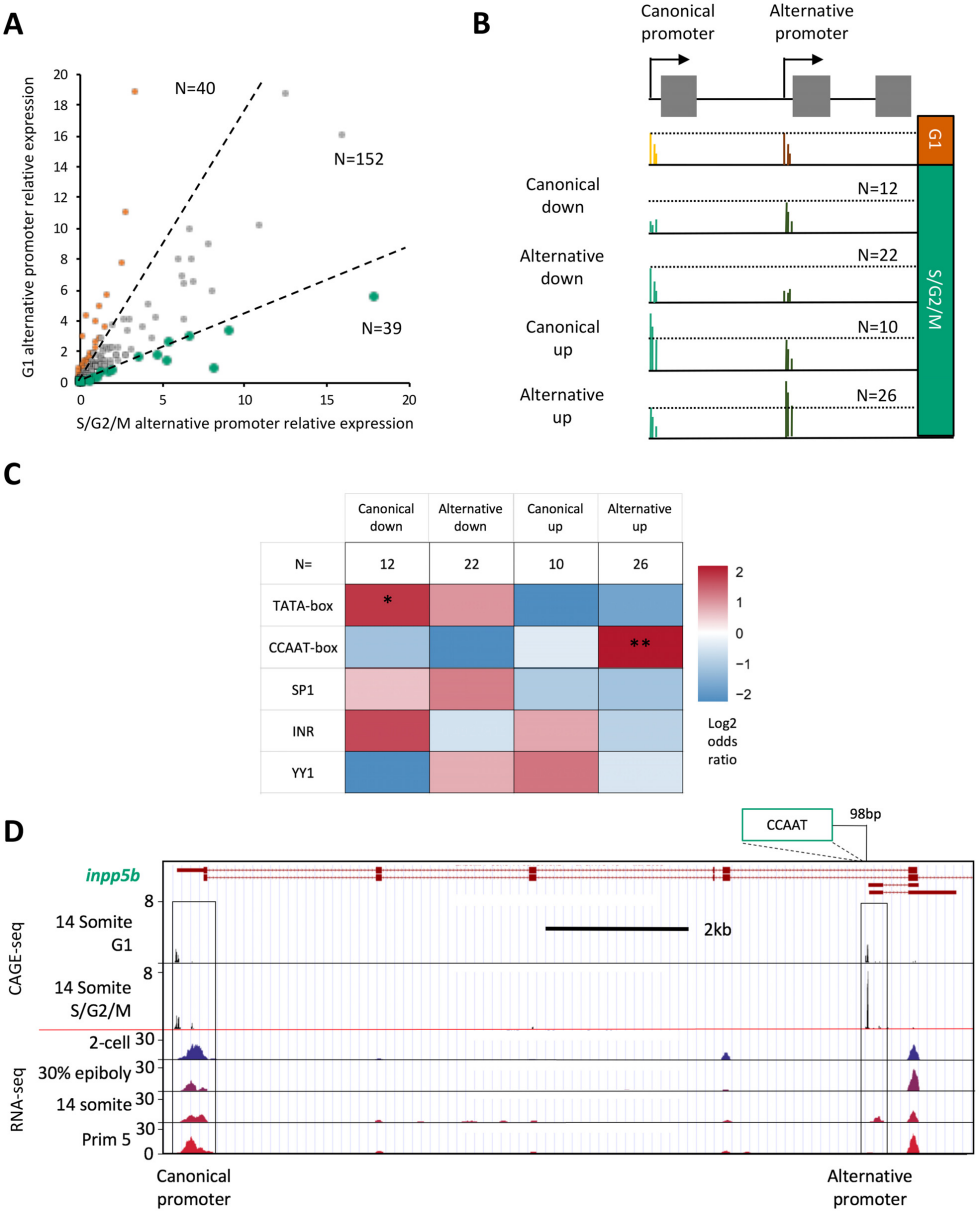
Alternative promoter usage. TPM values were determined for genomic regions identified in Nepal *et al.* (31) to correspond to canonical and alternative promoters, associated with the same genes (*n* = 1612). Genes with significant expression (TPM > 5 in either the canonical or alternative promoter region in both the G1 and S/G2/M populations) were taken for further analysis (*n* = 231). (**A**) Plot of correlation of alternative promoter utilization, normalized to canonical promoter expression, between G1 and S/G2/M populations. Dashed lines denote threshold of 2-fold change in normalized alternative promoter expression between the populations (*n* = 79). These differentially utilized genes were segregated by whether a change in the expression of the canonical or alternative promoter, in either population, was responsible for the 2-fold change in the expression of the alternative promoter, normalized to canonical promoter expression. (**B**) Diagrammatic summary of group selection criteria. In brief, relative to the G1 population; *Canonical down*: S/G2/M alternative promoter expression unchanged, but canonical expression depleted (*n* = 12); *Alternative down*: S/G2/M alternative promoter expression depleted, but canonical expression unchanged (*n* = 22); *Canonical up*: S/G2/M alternative promoter expression unchanged, but canonical expression enhanced (*n* = 10); *Alternative up*: S/G2/M alternative promoter expression enhanced, but canonical expression unchanged. Situations falling outside of these criteria were discarded (n = 9). Full selection criteria shown in materials and methods. (**C**) Heatmap visualizing the log2 odds ratio of selected promoter motif occurrence for each group. Occurrence is scored in each group vs. occurrence in the rest of the data set (*n* = 80) (*P< 0.05, **P< 0.01, ***P< 0.001, Fisher's exact test). (**D**) UCSC genome browser view of the *inositol polyphosphate-5-phosphatase B* (*inpp5b)* promoters with CAGE-seq tracks showing enhanced alternative promoter usage in the S/G2/M population versus G1, an example of a member of ‘Alternative up’ group. The position of a CCAAT motif relative to the alternative promoter is shown. RNA-seq tracks, imported from the ‘Promoterome CAGE and nucleosome positioning’ publicly available trackhub (URL: http://trackhub.genereg.net/promoterome/danRer7/index.html) (17,31) show that this alternative promoter drives a somitogenesis specific transcript.

A major source of divergence in the transcriptome of cell populations, particularly during differentiation, is through the use of alternative promoters (31). In order to investigate whether the regulatory differences, observed with differentially expressed and promoter shape transitioning genes, also impact on the utilization of alternative promoters, the relative expression of previously identified alternative promoter containing genes in zebrafish (31), was determined between populations. TPM values were calculated for genomic regions identified to correspond to canonical and alternative promoters, associated with the same genes (*n* = 1612). Genes with significant expression (TPM > 5 in either the canonical or alternative promoter region, in both the G1 and S/G2/M populations) were selected for further analysis (*n* = 231). In order to identify genes with differential utilization of alternative promoters, alternative promoter TPM values were normalized to canonical (‘alternative promoter relative expression’) and compared between populations (Figure 6A). This analysis identified 79 genes (34% of significantly expressed candidates) with a 2-fold change in the relative expression of the alternative promoter.

In order to determine which of these relative changes in expression were due to an upregulation in the canonical or alternative promoter in either the G1 or S/G2/M populations, these genes were subdivided into four groups as detailed in Figure 6B. They are as follows; relative to the G1 population; *Canonical down*: S/G2/M alternative promoter expression is unchanged, but canonical expression is depleted (*n* = 12); *Alternative down*: S/G2/M alternative promoter expression is depleted, but canonical expression is unchanged (*n* = 22); *Canonical up*: S/G2/M alternative promoter expression is unchanged, but canonical expression is enhanced (*n* = 10); *Alternative up*: S/G2/M alternative promoter expression is enhanced, but canonical expression is unchanged. Situations falling outside of these criteria were discarded (*n* = 9). Full selection criteria are detailed in the materials and methods. In order to determine whether, like differentially expressed and shape changing promoters, these alternative promoter utilization events are marked by differential regulatory machinery to each other, promoter proximal regulatory motif analysis was performed as before on the promoter, where the change in expression is occurring (Figure 6C). This analysis revealed a significant enrichment for TATA utilization in genes where the canonical promoter is depleted in S/G2/M (*P* < 0.05) and significant CCAAT utilization enrichment where the alternative promoter is enriched in S/G2/M (*P* < 0.01). An example of the latter is shown in Figure 6D. The utilization of the canonical promoter of *inositol polyphosphate-5-phosphatase B* (*inpp5b)* is unchanged between population, however expression from an alternative, CCAAT-box containing, promoter is enhanced in S/G2/M. Publicly available RNA-seq data from (17,31) suggests that this promoter drives an alternative segmentation period specific transcript from the *inpp5b* gene (Figure 6D). Further analysis of the canonical promoter of *inpp5b* also reveals that despite overall expression being unchanged, it harbors an S/G2/M specific isoform proximal to two CCAAT-boxes (Supplementary Figure S7). The RNA-seq data suggest this may also be a segmentation period specific isoform of *inpp5b*. This analysis further supports a role for differential TATA- and CCAAT-box core-promoter utilization in defining disparate transcriptional output, between populations segregated by cell cycle dynamic behaviour, during the segmentation period of embryo development.

### Genes with temporal expression dynamics during zebrafish embryonic segmentation are marked by differential promoter motif utilization and TSS distribution

As previously discussed, the zebrafish FUCCI system revealed a clear transition from a predominantly rapidly cycling cell population in early segmentation stages, to an increasing predominance of slowly cycling cells in late segmentation/early pharyngula stages (Supplementary Figure S1A, movie 1, (21)). We therefore wanted to determine whether the cell cycle associated transitions in promoter utilization, demonstrated in this study, are specific to the 14-somite stage of zebrafish development, or rather reflect a transition in promoter behaviour over the segmentation period, marked by cell cycle dynamic changes. In order to investigate this, we used previously published CAGE data from the 4-somite, 14-somite and Prim-5 stages of zebrafish development (30,31). This data was processed and analysed in the same manner described previously, to generate a dataset where promoter behaviour can be compared between segmentation stages.

**Figure 7.**
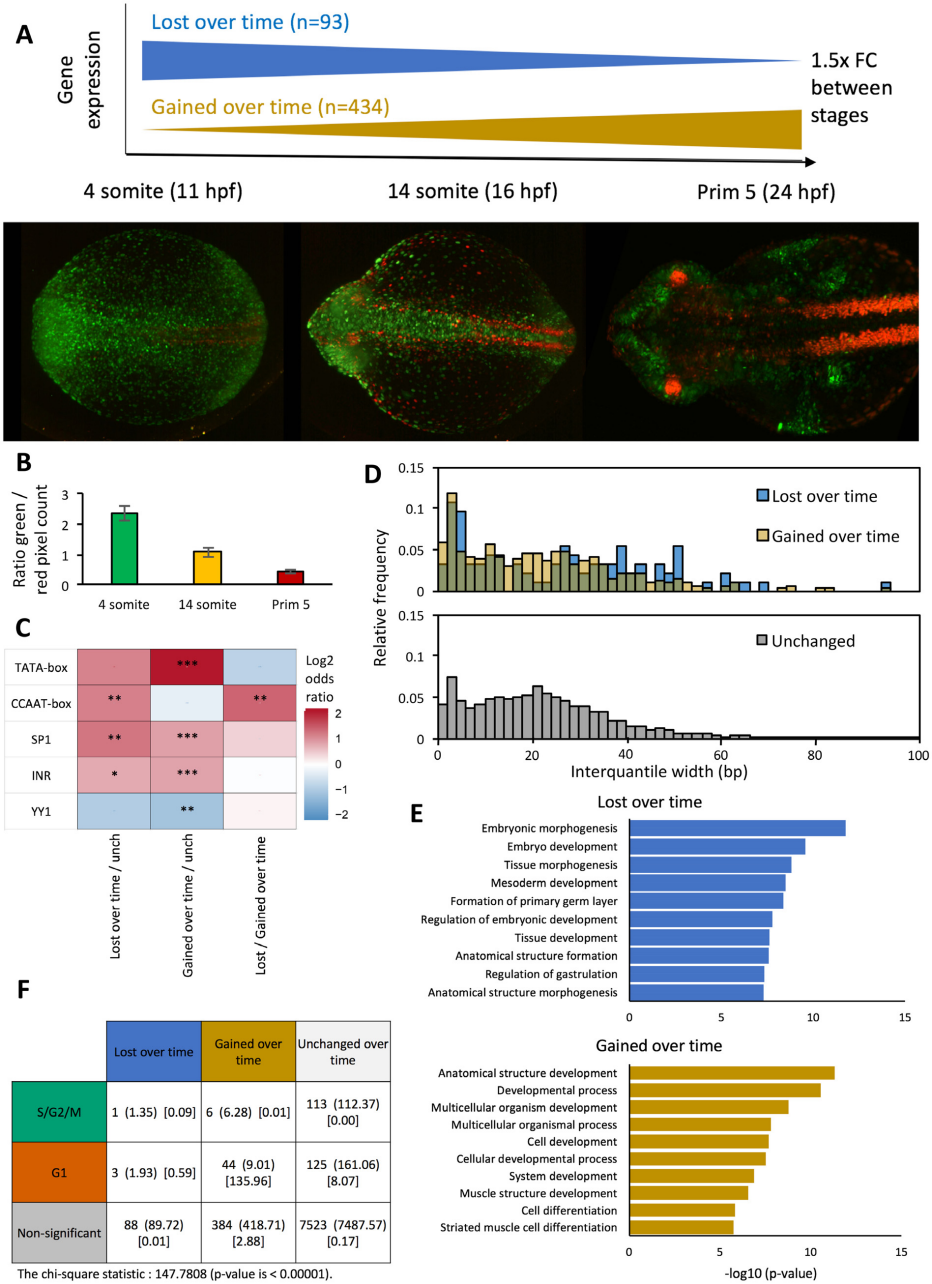
Genes with opposing temporal expression dynamics are marked by differential promoter motif utilization and TSS distribution. Published CAGE data from (30,31) was processed to generate a differential expression and promoter behaviour matrix for 4-somite, 14-somite and prim-5, three stages of zebrafish segmentation with differing ratios of rapid to slowly cycling cells, as demonstrated by Fucci imaging (A) and fluorophore pixel ratios (B). (**A**) Relative expression analysis of consensus clusters with a TPM > 5 in at least one time stage was performed. Genes where expression changes in the same direction by >1.5 fold in each sequential time stage were selected for further analysis and divided into gene where expression is lost over time (n = 93) and gained over time (n = 434) (4 somite → 14 somite [11–16 h post fertilization]→ Prim 5 [24hpf]). (**B**) Bar graph showing ratio of green / red fluorophore pixel density from imaged Fucci embryos at 4 and 14 somite and Prim 5 stages. (**C**) Heatmap visualizing the log_2_ odds ratio of selected promoter motif occurrence for genes with expression lost, or gained over time versus genes with no temporal dynamics (*n* = 7762) (*P< 0.05, **P< 0.01, ***P< 0.001, Fisher's exact test). (**D**) Consensus cluster interquantile width (IQW) in genes with expression lost (blue, mean IQW: 27.5), gained (yellow, mean IQW: 23.0) over time, or no temporal expression dynamics (grey, mean IQW: 22.8), visualized as a histogram. IQW was taken from the developmental stage where consensus cluster expression was highest. (**E**) Gene ontology analysis of genes with expression lost / gained over time. (**F**) Table showing chi-square intersection analysis between gene sets with temporal dynamics over the segmentation period of zebrafish development and cell cycle dynamics during the 14 somite stage. Data format: expected intersection size (chi-square statistic).

In order to confirm the selected stages have suitably distinct cell cycle dynamic behaviour, FUCCI transgenic embryos from the selected stages were imaged and the ratio of green (rapid cycling) to red (slow cycling) fluorescence pixel density was measured (Figure 7A and B). This analysis revealed a clear reduction in the proportion of rapidly to slowly cycling cells over time, with clear distinctions in the proportions from the selected stages (2.4:1 [4-somite], 1.1:1 [14-somite], 0.4:1 [Prim-5], *n* = 10 for each stage).

Analysis of the multi-segmentation-stage CAGE revealed that 8130 consensus clusters were shared between the 4-somite, 14-somite and Prim-5 stages. Comparative analysis of TPM values in each sample revealed two populations of genes with temporal expression dynamics over the segmentation period of embryo development. These are those with expression lost over time (1.5-fold expression level change between each stage, 4-somite > 14-somite > Prim-5, *n* = 93) and those with expression gained over time (4-somite < 14-somite < Prim-5, n = 434) (Figure 7A). Analysis of promoter motif utilization revealed a significant enrichment in the utilization of the CCAAT-box in genes with expression lost over time (*P* < 0.01), as the embryo transitions from a predominance of rapid to slowly cycling cells, in agreement with previous analyses in this study (Figure 7C). Conversely, genes with expression gained over time were significantly enriched for TATA-box utilization (*P* < 0.001), again in agreement with this study's data on motif association with cell cycle dynamics. Interestingly Sp1 motif utilization, previously shown to be associated with slowly cycling cells, was enriched in both gene sets relative to genes with unchanged temporal expression, suggesting some divergence in the gene sets selected by temporal versus cell cycle dynamics. Assessment of promoter interquantile widths in these gene sets revealed a subtle, but measurable broadening of TSS distribution in genes lost versus gained over time (average IQW = 27.5 versus 23) (Figure 7D). It is not as decisive as in the gene set selected by cell cycle dynamics, but follows the same pattern.

Comparison of gene ontology between the two gene sets revealed a remarkable overlap in terms associated with slowly cycling cells and genes with expression gained over time, both being strongly associated with differentiation, particularly of muscle tissues. There was considerably more divergence between terms associated with rapidly cycling cells and those with expression lost over the segmentation period, the latter being associated with the regulation of morphogenesis and gastrulation, which has just finished at the 4-somite stage, rather than the regulation of cell proliferation marking rapidly cycling cells (Figure 7E). In agreement with this, Chi-square analysis of temporal and cell cycle dynamic gene set overlap revealed a highly significant association (chi-square statistic: 135.96, *P* < 0.00001) between genes enriched in slowly cycling cells and those with expression gained over the segmentation period of embryo development (Figure 7F). The promoter behaviour of both these gene sets was defined by enhanced TATA utilization and narrow TSS distribution. There was no significant overlap between the gene sets enriched in rapidly cycling cells and with expression lost over the segmentation period. Strikingly however both are characterized by enhanced CCAAT-box utilization and broadening of TSS distribution. These finding suggest that the divergence in promoter behaviour described in this study does not only reflect differentiation associated changes in cell cycle dynamics occurring at the mid-segmentation stage (14-somite), but also marks a temporal switch in gene expression behaviour occurring over this period of embryonic segmentation.

## DISCUSSION

The developing zebrafish embryo displays extensive transitioning in cell cycle dynamics associated with cell differentiation. Previous studies have described how this process is accompanied by changes in transcriptional output, but this study is the first to show the role the core-promoter plays in defining this output through local regulatory changes. In this investigation, cells from segmentation period FUCCI transgenic embryos were dissociated and segregated by cell cycle stage (G1 versus S/G2/M). Differential expression analysis on these populations revealed a striking separation in the identity of these cells. Beside differences in cell cycle stage, slowly cycling (G1) cells showed extensive specification in terms of gene expression to different tissues, associated with increased representation of tissue-specific regulatory motifs in their promoters. Rapidly cycling (S/G2/M) cells, on the other hand, were far less specified, with overwhelming representation of ubiquitously expressed genes, associated with DNA and chromatin processing required for rapid proliferation. This divergence in cellular identity was found to be concurrent with changes in promoter behaviour. Differentially expressed genes were sharper in the G1 population and associated with an increase in TATA-box utilization, alongside Sp1 binding sites. In contrast, the broader promoter usage in S/G2/M upregulated genes was accompanied by a far greater utilization of CCAAT-box general transcription factor binding sites and a lesser dependence on TATA. TATA-like and W-box motif frequency was found to be enriched in rapidly cycling cells however. These changes in promoter behaviour were found to be predominantly associated with differential cell cycle behaviour, rather than tissue lineage identity. In addition, global analysis of changes in promoter associated TSS distribution (shape change) and usage of alternative promoters, revealed these again to be associated with divergent utilization of TATA and CCAAT-box TF binding sites. Additionally, this pattern of promoter behaviour was found to be replicated in genes with temporal changes in expression over the segmentation period of zebrafish embryo development, a stage at which there is extensive transitioning in cell cycle dynamics.

Collectively this data suggests a strong divergence in the utilization of general transcription factor binding sites, TATA and CCAAT, as well as Sp1 binding sites, between cells undergoing differentiation coupled changes in cell cycle dynamics. This divergence impacts on the transcriptome and promoterome of these cells, resulting in differential gene expression as well as differential canonical and alternative promoter utilization.

Promoter proximal regulatory elements have previously been associated with marking genes with cell cycle periodic expression. The promoter-associated cell cycle-dependent element (CDE) and the cell cycle genes homology region (CHR) have both been found to regulate genes with maximum expression in G2-M phase, through cell cycle stage dependent binding of transcription factors (reviewed in (43)). CCAAT-boxes have also been found to play a role, in association with these factors. Three CCAAT-boxes, along with a single cell cycle gene homology region (CHR), were found to be major regulatory sites for the transcription of human cyclin B2, through NF-Y binding (44). Additionally CCAAT/enhancer binding protein β (C/EBPβ) was found to be a key driver of stromal cell progress through the cell cycle (45). This suggests that promoter based regulatory signalling is key to controlling gene expression cell cycle periodicity. This study extends this by displaying a role for CCAAT-boxes in genes differentially regulated during differentiation coupled changes in cell cycle dynamics, associated with broad TSS distribution across the promoter.

This study shows that cells going through the striking changes in cell cycle dynamics, occurring embryo wide, during the segmentation period, have differential tissue-specification. This may suggest a marked transition in multiple tissue progenitors during this process. Rapidly cycling cells, show weak tissue-specificity despite their clear segregation to distinct tissue domains such as the eye cup, notochord and brain tissues. Additionally, they show a transcriptional profile dominated by ubiquitously expressed DNA processing genes and cell cycle regulators (46). This population of genes has been found to be downregulated at the onset of organogenesis in mouse and shortly after gastrulation in drosophila (47), highlighting the modulation of this class of genes as a key marker of tissue-specification. Slowly cycling cells on the other hand show a far more tissue-specified transcriptional program, linked with a greater utilization of the TATA-box associated gene regulatory machinery.

Single cell RNA-seq analysis of early stage embryo development (from high stage to 6-somite) does reveal an early specification point, differentiating notochord and brain tissues (both rapidly cycling) from somites and cardiac tissues (both slowly cycling) (48). This data supports the idea that the spatial segregation of cells based on cell cycle dynamics is associated with lineage-specification. As stated however, this analysis only extended to the 6-somite stage, when the vast majority of cells are rapidly cycling (Supplementary Figure S1A). In combination with the findings of this investigation, this data suggests that while lineages are specified early in development by small transcriptional changes (but with cell cycle and DNA processing genes predominant and no discernable cell cycle dynamic changes), it is at the point of tissue differentiation that a major shift in transcriptional output occurs, associated with an elongation of the G1 phase. In the future, combining the approaches used in both investigations, possibly using newly developed single cell CAGE protocols (such as C1-CAGE) (49) will permit this process to be further explored.

Investigations into differentiation associated changes in gene regulatory programs, from terminally differentiated gamete, to pluripotent stem cell (17) and lineage defined multipotent stem cell, to terminally differentiated tissue cell (5,22), have also found dramatic changes in gene regulatory program. These have been associated with changes in the utilization of TATA, TATA-like and W-box machinery (5,17,22). Terminally differentiated gametes reprogram to pluripotent cells in the early embryo, marked by W-box restricted programs being replaced with open CpG island associated promoter utilization, restricted only by +1 nucleosome positioning. This potentially primes pluripotent cells for a more diversified repertoire of behaviour as their lineage is specified (17). This process is also marked by a transition from rapid synchronous cell cycles, to slower asynchronous cycling, in zebrafish embryos. In this paper we show that cells then slow their cycling to differentiate and defining tissues as the body map starts to form, and this process is marked by an upregulation of TATA driven expression of tissue-specific genes and down regulation of ubiquitously expressed DNA and chromatin processing machinery. Studies, investigating transitions from lineage defined multipotent stem cells to terminally differentiated cells in both muscle (22) and liver development (5) have shown that this process is marked by wholesale depletion of RNA polymerase II regulatory cofactors, in particular TATA-associated factors. This is accompanied by a restriction to a small cohort of functional regulatory elements, leading to a limitation of the transcriptional repertoire, to be highly specialized and tissue-specific, in terminally differentiated cells. Collectively, this suggests that differentiation transitions in embryonic development are intimately associated with promoter level changes in the gene regulatory program, with the TATA-box a key component.

The TATA-box has previously been characterized as playing a crucial role in defining the tissue-specificity of associated genes, with precise TATA-TSS spacing a vital factor (8). Additionally, tissue-specific genes in *D. melanogaster* and mammalian systems are often associated with the presence of a promoter proximal, spatially constrained TATA-boxes, alongside sharp transcription initiation, differentiating this class of promoter (type I) from non-TATA dependent housekeeping (type II) or developmentally regulated (type III) gene associated promoters (12,50–52). This paper extends the tissue-specification/TATA-box association story by showing that an increase in TATA-box utilization is one of the major defining factors in specifying differentiating cells at the earliest stages of tissue-lineage-specification.

Interestingly, in contrast to TATA-box utilization, TATA-like and W-box motifs were found to be enriched proximal to the promoters of genes upregulated in rapidly cycling cells. TATA-like elements have previously been associated with more dispersed TSS distribution on promoters, in particular multi-modal promoters (two or more dominant TSSs in the promoter) (8). Additionally, transcription mediated by TATA-like motifs had been found to predominantly regulated by the TFIID complex, rather than SAGA, in contrast to TATA-box regulation, in yeast (53). The regulatory distinction between TFIID and SAGA dominant genes has subsequently been challenged however, with SAGA reported to be a general cofactor required for virtually all RNA polymerase II transcription (54). This dependence on TFIID has been found to be subject to the presence of additional promoter regulatory elements including the upstream activating sequence (UAS) (55). The SAGA complex has not been identified in vertebrates, so how transferable this observation is to zebrafish development is uncertain, however it does demonstrate the TATA and TATA-like elements confer distinct regulatory identities to the promoters in which they reside. Significant and contrasting differences in the utilization of these motifs, identified in this study, in genes differentially expressed in fast and slowly cycling cells, suggests distinct promoter level regulatory programs activated between these populations.

Sp1 has also been associated with regulating tissue-specification, in particular through interaction with cell cycle regulated factors (56). Sp1 has been implicated in the transactivation of differentiation-regulated genes, underlying the switch from proliferation to differentiation, in squamous epithelium, through interactions with cell cycle regulators, retinoblastoma protein (pRB) and cyclin D1 (57). Alongside this, Sp1 has been identified as a key regulator of the G1 phase of the cell cycle in epithelial cells (58). This paper extends these observations by demonstrating the activity of Sp1 in the regulation of genes involved in the transition of cells, from a proliferation to differentiation phenotype in a developing *in vivo* model.

This investigation demonstrates for the first time the promoter level regulatory changes occurring in cells undergoing the transition from proliferation to tissue-specification, at the segmentation stage of embryo development, *in vivo*. It displays how this process is coupled with transitions in cell cycle dynamics to a slower cycling rate where the G1-phase predominates, alongside transitions in promoter behaviour. This is marked by a sharpening of promoter utilization, from rapidly to slowly cycling cells, mediated by increased utilization of TATA-box and Sp1 regulatory units, at the expense of the CCAAT-box. This transition in the promoterome is reflected in the transcriptome where the predominance of DNA and chromatin processing factors in rapidly cycling unspecified cells is replaced by increased expression of tissue-lineage specifying genes.

## DATA AVAILABILITY

Sequencing files for this project can be found at the ArrayExpress accession: E-MTAB-8795.

## Supplementary Material

gkaa563_Supplemental_Files

## References

[1] Filipczyk A.A., Laslett A.L., Mummery C., Pera M.F. Differentiation is coupled to changes in the cell cycle regulatory apparatus of human embryonic stem cells. Stem Cell Res. 2007; 1:45–60. 19383386 10.1016/j.scr.2007.09.002

[2] Ohtsuka S., Dalton S. Molecular and biological properties of pluripotent embryonic stem cells. Gene Ther. 2008; 15:74–81. 17989701 10.1038/sj.gt.3303065

[3] Calder A., Roth-Albin I., Bhatia S., Pilquil C., Lee J.H., Bhatia M., Levadoux-Martin M., McNicol J., Russell J., Collins T. et al. Lengthened G1 phase indicates differentiation status in human embryonic stem cells. Stem Cells Dev. 2013; 22:279–295. 22827698 10.1089/scd.2012.0168

[4] Roccio M., Schmitter D., Knobloch M., Okawa Y., Sage D., Lutolf M.P. Predicting stem cell fate changes by differential cell cycle progression patterns. Development. 2013; 140:459–470. 23193167 10.1242/dev.086215

[5] D’Alessio J.A., Ng R., Willenbring H., Tjian R. Core promoter recognition complex changes accompany liver development. Proc. Natl. Acad. Sci. U.S.A. 2011; 108:3906–3911. 21368148 10.1073/pnas.1100640108 PMC3054039

[6] Kadonaga J.T. Perspectives on the RNA polymerase II core promoter. Wiley Interdiscip. Rev. Dev. Biol. 2012; 1:40–51. 23801666 10.1002/wdev.21 PMC3695423

[7] Carninci P., Sandelin A., Lenhard B., Katayama S., Shimokawa K., Ponjavic J., Semple C.A., Taylor M.S., Engstrom P.G., Frith M.C. et al. Genome-wide analysis of mammalian promoter architecture and evolution. Nat. Genet. 2006; 38:626–635. 16645617 10.1038/ng1789

[8] Ponjavic J., Lenhard B., Kai C., Kawai J., Carninci P., Hayashizaki Y., Sandelin A. Transcriptional and structural impact of TATA-initiation site spacing in mammalian core promoters. Genome Biol. 2006; 7:R78. 16916456 10.1186/gb-2006-7-8-r78 PMC1779604

[9] Sandelin A., Carninci P., Lenhard B., Ponjavic J., Hayashizaki Y., Hume D.A. Mammalian RNA polymerase II core promoters: insights from genome-wide studies. Nat. Rev. Genet. 2007; 8:424–436. 17486122 10.1038/nrg2026

[10] Lenhard B., Sandelin A., Carninci P. Metazoan promoters: emerging characteristics and insights into transcriptional regulation. Nat. Rev. Genet. 2012; 13:233–245. 22392219 10.1038/nrg3163

[11] Juven-Gershon T., Kadonaga J.T. Regulation of gene expression via the core promoter and the basal transcriptional machinery. Dev. Biol. 2010; 339:225–229. 19682982 10.1016/j.ydbio.2009.08.009 PMC2830304

[12] Akalin A., Fredman D., Arner E., Dong X., Bryne J.C., Suzuki H., Daub C.O., Hayashizaki Y., Lenhard B. Transcriptional features of genomic regulatory blocks. Genome Biol. 2009; 10:R38. 19374772 10.1186/gb-2009-10-4-r38 PMC2688929

[13] Plessy C., Pascarella G., Bertin N., Akalin A., Carrieri C., Vassalli A., Lazarevic D., Severin J., Vlachouli C., Simone R. et al. Promoter architecture of mouse olfactory receptor genes. Genome Res. 2012; 22:486–497. 22194471 10.1101/gr.126201.111 PMC3290784

[14] Deaton A.M., Bird A. CpG islands and the regulation of transcription. Genes Dev. 2011; 25:1010–1022. 21576262 10.1101/gad.2037511 PMC3093116

[15] Kimmel C.B., Ballard W.W., Kimmel S.R., Ullmann B., Schilling T.F. Stages of embryonic development of the zebrafish. Dev. Dyn. 1995; 203:253–310. 8589427 10.1002/aja.1002030302

[16] Wragg J., Muller F. Transcriptional regulation during zygotic genome activation in zebrafish and other anamniote embryos. Adv. Genet. 2016; 95:161–194. 27503357 10.1016/bs.adgen.2016.05.001

[17] Haberle V., Li N., Hadzhiev Y., Plessy C., Previti C., Nepal C., Gehrig J., Dong X., Akalin A., Suzuki A.M. et al. Two independent transcription initiation codes overlap on vertebrate core promoters. Nature. 2014; 507:381–385. 24531765 10.1038/nature12974 PMC4820030

[18] Nestorov P., Hotz H.R., Liu Z., Peters A.H. Dynamic expression of chromatin modifiers during developmental transitions in mouse preimplantation embryos. Sci. Rep. 2015; 5:14347. 26403153 10.1038/srep14347 PMC4585904

[19] Penalosa-Ruiz G., Bright A.R., Mulder K.W., Veenstra G.J.C. The interplay of chromatin and transcription factors during cell fate transitions in development and reprogramming. Biochim. Biophys. Acta Gene Regul. Mech. 2019; 1862:194407. 31356991 10.1016/j.bbagrm.2019.194407

[20] Sakaue-Sawano A., Kurokawa H., Morimura T., Hanyu A., Hama H., Osawa H., Kashiwagi S., Fukami K., Miyata T., Miyoshi H. et al. Visualizing spatiotemporal dynamics of multicellular cell-cycle progression. Cell. 2008; 132:487–498. 18267078 10.1016/j.cell.2007.12.033

[21] Sugiyama M., Sakaue-Sawano A., Iimura T., Fukami K., Kitaguchi T., Kawakami K., Okamoto H., Higashijima S., Miyawaki A. Illuminating cell-cycle progression in the developing zebrafish embryo. Proc. Natl. Acad. Sci. U.S.A. 2009; 106:20812–20817. 19923430 10.1073/pnas.0906464106 PMC2779202

[22] Deato M.D., Tjian R. Switching of the core transcription machinery during myogenesis. Genes Dev. 2007; 21:2137–2149. 17704303 10.1101/gad.1583407 PMC1950853

[23] Muller F., Tora L. Chromatin and DNA sequences in defining promoters for transcription initiation. Biochim. Biophys. Acta. 2014; 1839:118–128. 24275614 10.1016/j.bbagrm.2013.11.003

[24] Haberle V., Stark A. Eukaryotic core promoters and the functional basis of transcription initiation. Nat. Rev. Mol. Cell Biol. 2018; 19:621–637. 29946135 10.1038/s41580-018-0028-8 PMC6205604

[25] Levine M., Cattoglio C., Tjian R. Looping back to leap forward: transcription enters a new era. Cell. 2014; 157:13–25. 24679523 10.1016/j.cell.2014.02.009 PMC4059561

[26] Westerfield M. The Zebrafish Book: A Guide for the Laboratory Use of Zebrafish (Danio Rerio). 2000; University of Oregon Press.

[27] Schneider C.A., Rasband W.S., Eliceiri K.W. NIH Image to ImageJ: 25 years of image analysis. Nat. Methods. 2012; 9:671–675. 22930834 10.1038/nmeth.2089 PMC5554542

[28] Poulain S., Kato S., Arnaud O., Morlighem J.E., Suzuki M., Plessy C., Harbers M. NanoCAGE: A method for the analysis of coding and Noncoding 5'-Capped transcriptomes. Methods Mol. Biol. 2017; 1543:57–109. 28349422 10.1007/978-1-4939-6716-2_4

[29] Kuhn R.M., Karolchik D., Zweig A.S., Wang T., Smith K.E., Rosenbloom K.R., Rhead B., Raney B.J., Pohl A., Pheasant M. et al. The UCSC genome browser Database: update 2009. Nucleic Acids Res. 2009; 37:D755–D761. 18996895 10.1093/nar/gkn875 PMC2686463

[30] Nepal C., Hadzhiev Y., Balwierz P., Tarifeno-Saldivia E., Cardenas R., Wragg J.W., Suzuki A.M., Carninci P., Peers B., Lenhard B. et al. Dual-initiation promoters with intertwined canonical and TCT/TOP transcription start sites diversify transcript processing. Nat. Commun. 2020; 11:168. 31924754 10.1038/s41467-019-13687-0 PMC6954239

[31] Nepal C., Hadzhiev Y., Previti C., Haberle V., Li N., Takahashi H., Suzuki A.M., Sheng Y., Abdelhamid R.F., Anand S. et al. Dynamic regulation of the transcription initiation landscape at single nucleotide resolution during vertebrate embryogenesis. Genome Res. 2013; 23:1938–1950. 24002785 10.1101/gr.153692.112 PMC3814893

[32] Langmead B., Trapnell C., Pop M., Salzberg S.L. Ultrafast and memory-efficient alignment of short DNA sequences to the human genome. Genome Biol. 2009; 10:R25. 19261174 10.1186/gb-2009-10-3-r25 PMC2690996

[33] Haberle V., Forrest A.R., Hayashizaki Y., Carninci P., Lenhard B. CAGEr: precise TSS data retrieval and high-resolution promoterome mining for integrative analyses. Nucleic Acids Res. 2015; 43:e51. 25653163 10.1093/nar/gkv054 PMC4417143

[34] Balwierz P.J., Carninci P., Daub C.O., Kawai J., Hayashizaki Y., Van Belle W., Beisel C., van Nimwegen E. Methods for analyzing deep sequencing expression data: constructing the human and mouse promoterome with deepCAGE data. Genome Biol. 2009; 10:R79. 19624849 10.1186/gb-2009-10-7-r79 PMC2728533

[35] Yu G., Wang L.G., He Q.Y. ChIPseeker: an R/Bioconductor package for ChIP peak annotation, comparison and visualization. Bioinformatics. 2015; 31:2382–2383. 25765347 10.1093/bioinformatics/btv145

[36] Khan A., Fornes O., Stigliani A., Gheorghe M., Castro-Mondragon J.A., van der Lee R., Bessy A., Cheneby J., Kulkarni S.R., Tan G. et al. JASPAR 2018: update of the open-access database of transcription factor binding profiles and its web framework. Nucleic Acids Res. 2018; 46:D1284. 29161433 10.1093/nar/gkx1188 PMC5753202

[37] Adiconis X., Haber A.L., Simmons S.K., Levy Moonshine A., Ji Z., Busby M.A., Shi X., Jacques J., Lancaster M.A., Pan J.Q. et al. Comprehensive comparative analysis of 5'-end RNA-sequencing methods. Nat. Methods. 2018; 15:505–511. 29867192 10.1038/s41592-018-0014-2 PMC6075671

[38] Takahashi H., Kato S., Murata M., Carninci P. CAGE (cap analysis of gene expression): a protocol for the detection of promoter and transcriptional networks. Methods Mol. Biol. 2012; 786:181–200. 21938627 10.1007/978-1-61779-292-2_11 PMC4094367

[39] Kawaji H., Lizio M., Itoh M., Kanamori-Katayama M., Kaiho A., Nishiyori-Sueki H., Shin J.W., Kojima-Ishiyama M., Kawano M., Murata M. et al. Comparison of CAGE and RNA-seq transcriptome profiling using clonally amplified and single-molecule next-generation sequencing. Genome Res. 2014; 24:708–717. 24676093 10.1101/gr.156232.113 PMC3975069

[40] Carcamo J., Maldonado E., Cortes P., Ahn M.H., Ha I., Kasai Y., Flint J., Reinberg D. A TATA-like sequence located downstream of the transcription initiation site is required for expression of an RNA polymerase II transcribed gene. Genes Dev. 1990; 4:1611–1622. 2253881 10.1101/gad.4.9.1611

[41] Wang J., Xie Y., Bai X., Wang N., Yu H., Deng Z., Lian M., Yu S., Liu H., Xie W. et al. Targeting dual specificity protein kinase TTK attenuates tumorigenesis of glioblastoma. Oncotarget. 2018; 9:3081–3088. 29423030 10.18632/oncotarget.23152 PMC5790447

[42] Juven-Gershon T., Hsu J.Y., Kadonaga J.T. Perspectives on the RNA polymerase II core promoter. Biochem. Soc. Trans. 2006; 34:1047–1050. 17073747 10.1042/BST0341047

[43] Muller G.A., Engeland K. The central role of CDE/CHR promoter elements in the regulation of cell cycle-dependent gene transcription. FEBS J. 2010; 277:877–893. 20015071 10.1111/j.1742-4658.2009.07508.x

[44] Wasner M., Haugwitz U., Reinhard W., Tschop K., Spiesbach K., Lorenz J., Mossner J., Engeland K. Three CCAAT-boxes and a single cell cycle genes homology region (CHR) are the major regulating sites for transcription from the human cyclin B2 promoter. Gene. 2003; 312:225–237. 12909359 10.1016/s0378-1119(03)00618-8

[45] Wang W., Li Q., Bagchi I.C., Bagchi M.K. The CCAAT/enhancer binding protein beta is a critical regulator of steroid-induced mitotic expansion of uterine stromal cells during decidualization. Endocrinology. 2010; 151:3929–3940. 20501671 10.1210/en.2009-1437 PMC2940513

[46] Ramskold D., Wang E.T., Burge C.B., Sandberg R. An abundance of ubiquitously expressed genes revealed by tissue transcriptome sequence data. PLoS Comput. Biol. 2009; 5:e1000598. 20011106 10.1371/journal.pcbi.1000598 PMC2781110

[47] Fossat N., Pfister S., Tam P.P. A transcriptome landscape of mouse embryogenesis. Dev. Cell. 2007; 13:761–762. 18061558 10.1016/j.devcel.2007.11.011

[48] Farrell J.A., Wang Y., Riesenfeld S.J., Shekhar K., Regev A., Schier A.F. Single-cell reconstruction of developmental trajectories during zebrafish embryogenesis. Science. 2018; 360:eaar3131. 29700225 10.1126/science.aar3131 PMC6247916

[49] Kouno T., Moody J., Kwon A.T., Shibayama Y., Kato S., Huang Y., Bottcher M., Motakis E., Mendez M., Severin J. et al. C1 CAGE detects transcription start sites and enhancer activity at single-cell resolution. Nat. Commun. 2019; 10:360. 30664627 10.1038/s41467-018-08126-5 PMC6341120

[50] FitzGerald P.C., Sturgill D., Shyakhtenko A., Oliver B., Vinson C. Comparative genomics of Drosophila and human core promoters. Genome Biol. 2006; 7:R53. 16827941 10.1186/gb-2006-7-7-r53 PMC1779564

[51] Ohler U. Identification of core promoter modules in Drosophila and their application in accurate transcription start site prediction. Nucleic Acids Res. 2006; 34:5943–5950. 17068082 10.1093/nar/gkl608 PMC1635271

[52] Engstrom P.G., Ho Sui S.J., Drivenes O., Becker T.S., Lenhard B. Genomic regulatory blocks underlie extensive microsynteny conservation in insects. Genome Res. 2007; 17:1898–1908. 17989259 10.1101/gr.6669607 PMC2099597

[53] Rhee H.S., Pugh B.F. Genome-wide structure and organization of eukaryotic pre-initiation complexes. Nature. 2012; 483:295–301. 22258509 10.1038/nature10799 PMC3306527

[54] Baptista T., Grunberg S., Minoungou N., Koster M.J.E., Timmers H.T.M., Hahn S., Devys D., Tora L. SAGA is a general cofactor for RNA polymerase II transcription. Mol. Cell. 2017; 68:130–143. 28918903 10.1016/j.molcel.2017.08.016 PMC5632562

[55] Watanabe K., Kokubo T. SAGA mediates transcription from the TATA-like element independently of Taf1p/TFIID but dependent on core promoter structures in Saccharomyces cerevisiae. PLoS One. 2017; 12:e0188435. 29176831 10.1371/journal.pone.0188435 PMC5703507

[56] Tapias A., Ciudad C.J., Roninson I.B., Noe V. Regulation of Sp1 by cell cycle related proteins. Cell Cycle. 2008; 7:2856–2867. 18769160 10.4161/cc.7.18.6671 PMC2810142

[57] Opitz O.G., Rustgi A.K. Interaction between Sp1 and cell cycle regulatory proteins is important in transactivation of a differentiation-related gene. Cancer Res. 2000; 60:2825–2830. 10850422

[58] Grinstein E., Jundt F., Weinert I., Wernet P., Royer H.D. Sp1 as G1 cell cycle phase specific transcription factor in epithelial cells. Oncogene. 2002; 21:1485–1492. 11896576 10.1038/sj.onc.1205211

