# Supplementary material for: Embryonic tissue differentiation is characterized by transitions in cell cycle dynamic-associated core promoter regulation
Source: Nucleic Acids Res. 2020 Jul 3;48(15):8374–92. doi: 10.1093/nar/gkaa563 (PMC7470974; doi:10.1093/nar/gkaa563)
Supplement: gkaa563_Supplemental_Files [file gkaa563_supplemental_files.zip › Wragg et al., Supplementary figures and tables.pdf]

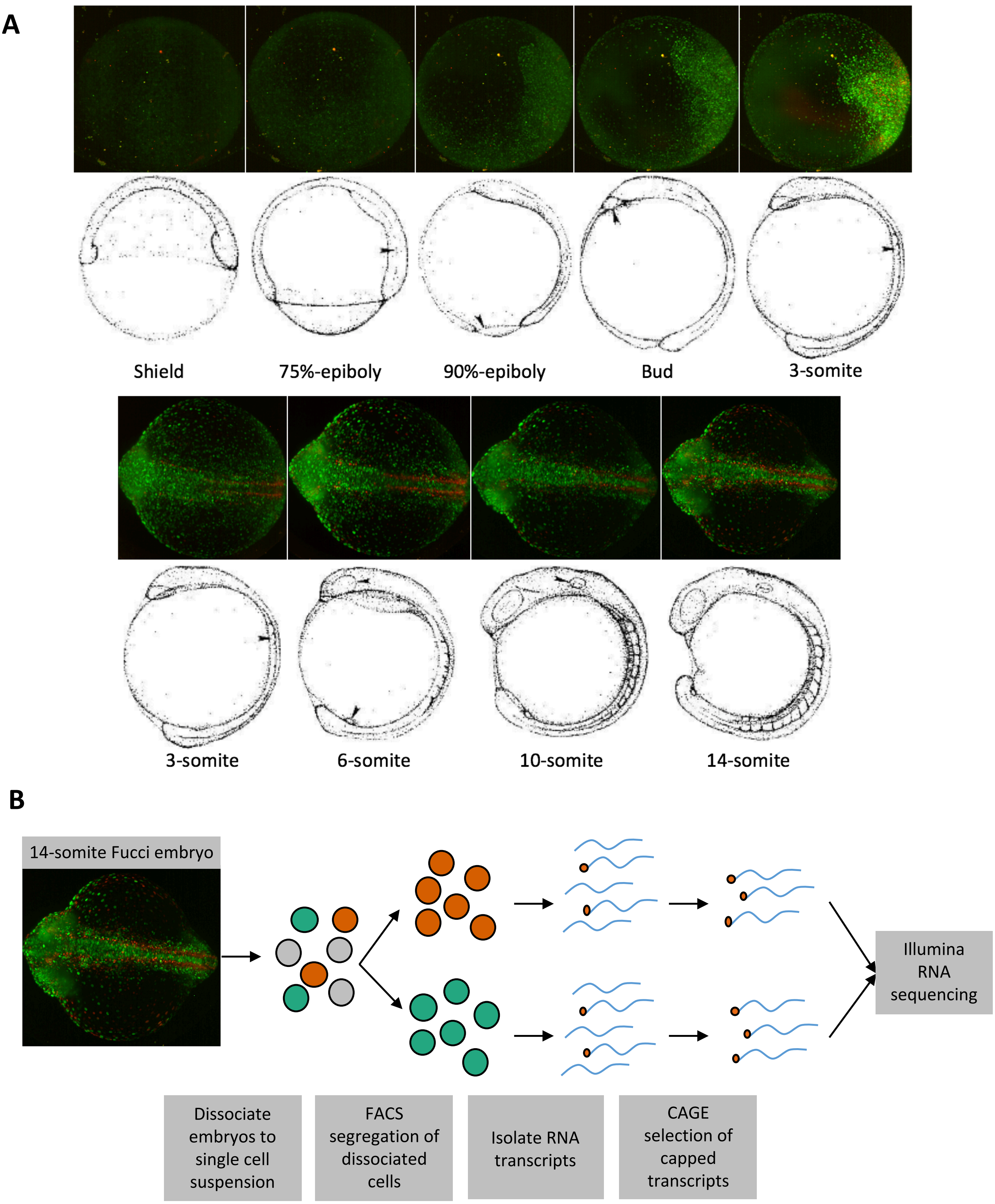

Supplementary figure 1. [A] Longitudinal analysis of FUCCI florescence in post-gastrulation embryos. Representative fluorescence images of FUCCI embryos from Shield to 14 somite stage. Drawings of embryo stage reproduced with permission from (15) [B] Flow diagram of experimental procedure for cycle-dynamics dependent segregation of cells and selection of capped transcripts for Illumina RNA sequencing and analysis of the promoterome.

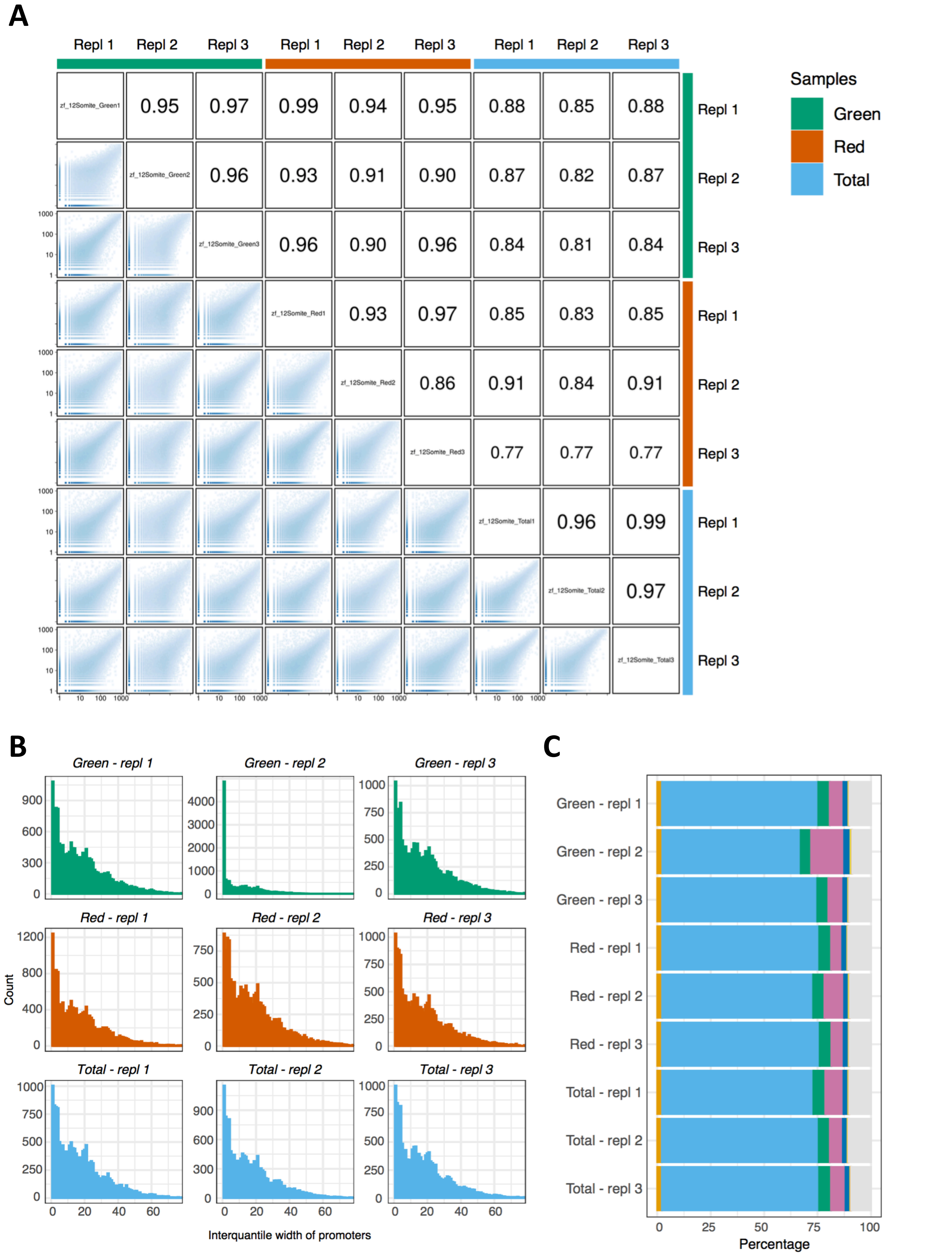

Supplementary figure 2. [A] CTSS correlation matrix for triplicate samples of G1 (Red), S/G2/M (Green) and unsegregated cells. [B] Graphs of tag cluster interquantile widths from the triplicate samples of G1 (Red), S/G2/M (Green) and unsegregated cells (Total). [C] Graph of mapping frequency to genomic features for triplicate samples of G1 (Red), S/G2/M (Green) and unsegregated cells. “Exon” (dark blue), “5’ UTR” (orange), “3’ UTR” (yellow) and “intron” (green) locations were extracted from the DanRer7 genomic build. “Promoter (<=1kb)” = window 0-1kb upstream of the gene start site (purple), “Promoter (1-3kb)” = 1-3kb region upstream of gene start (light blue), “Downstream <3kb” = window 0-3kb downstream of the gene end annotated in the DanRer7 genomic build (red), and “Distal intergenic” = all regions not covered in other classifications (grey).

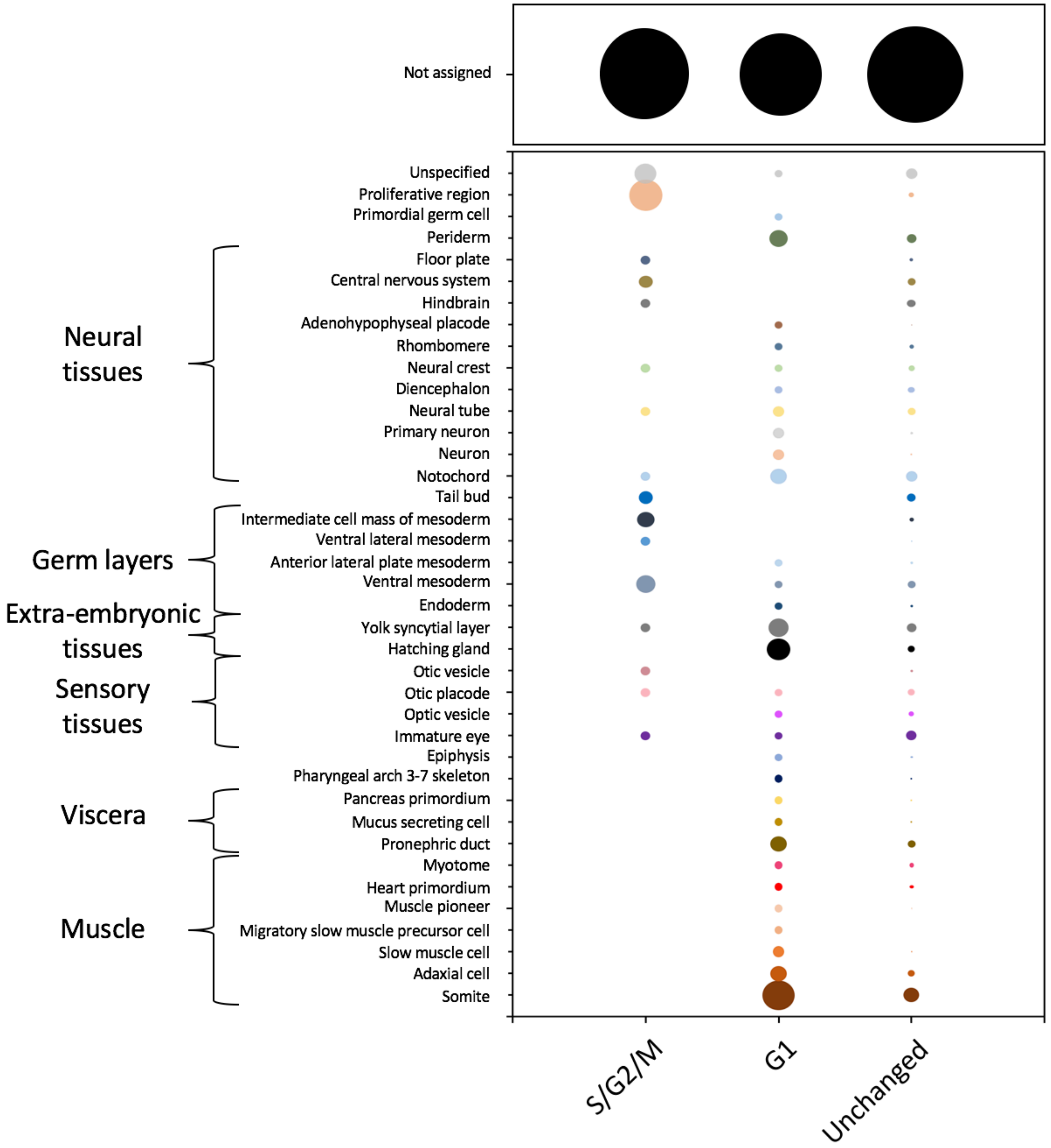

Supplementary figure 3. Bubble plot of tissue ontology of differentially expressed genes between S/G2/M and G1 cell populations (extracted from the ZFIN database of tissue specific gene expression for the 14-19 somite stage, https://zfin.org/downloads/wildtype-expression_fish.txt).

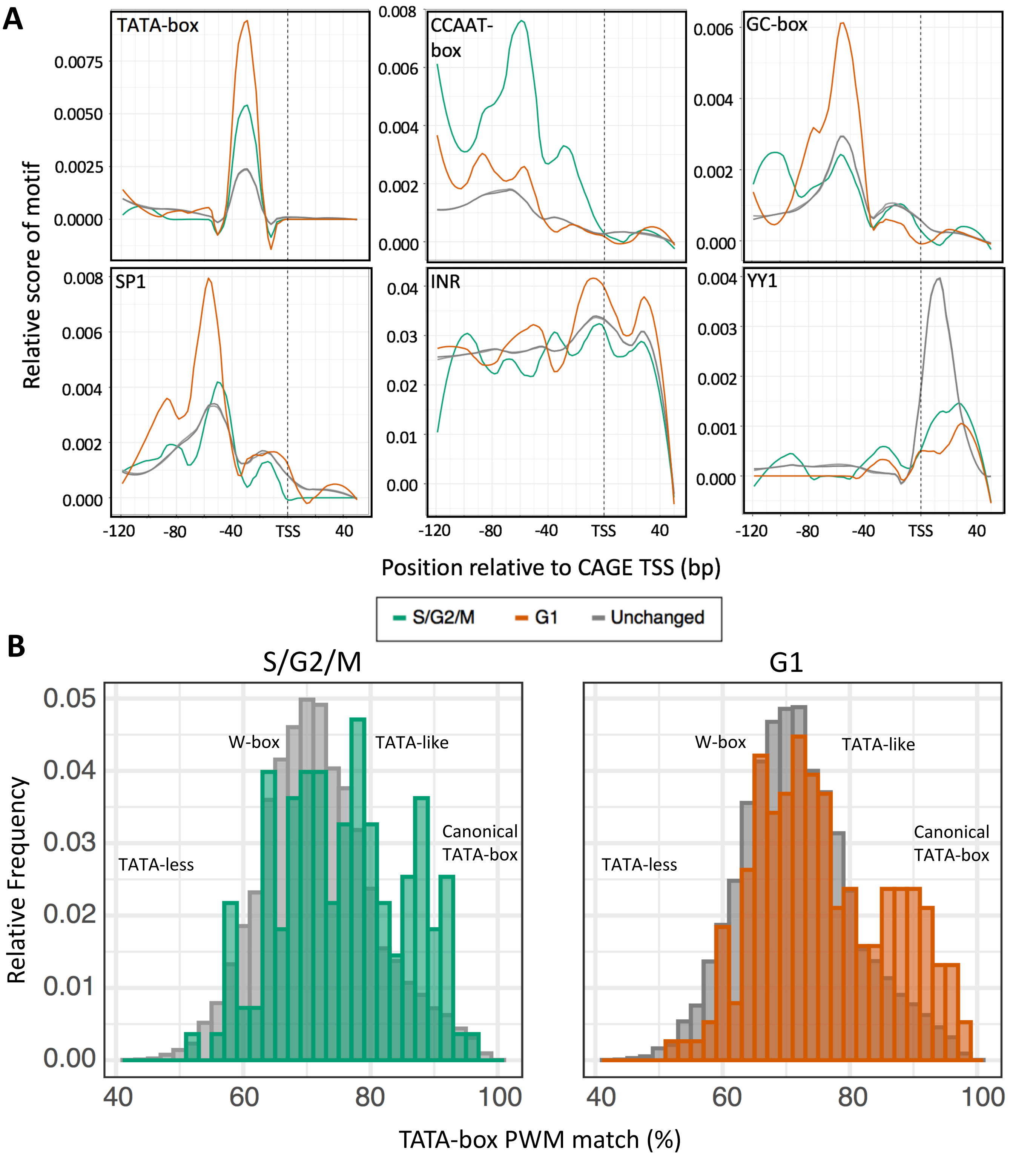

Supplementary figure 4. Additional core promoter architecture. [A] Metaplot of occurrence and positional constraint relative to TSS, of core promoter motifs (90% match) in different groups. [B] Distribution of position weight matrix (PWM) match (%) to TATA- box in the region –40 to –20 bp upstream of the dominant TSS in genes upregulated in S/G2/M (green) and G1 (orange) populations compared to genes with unchanged expression (grey).

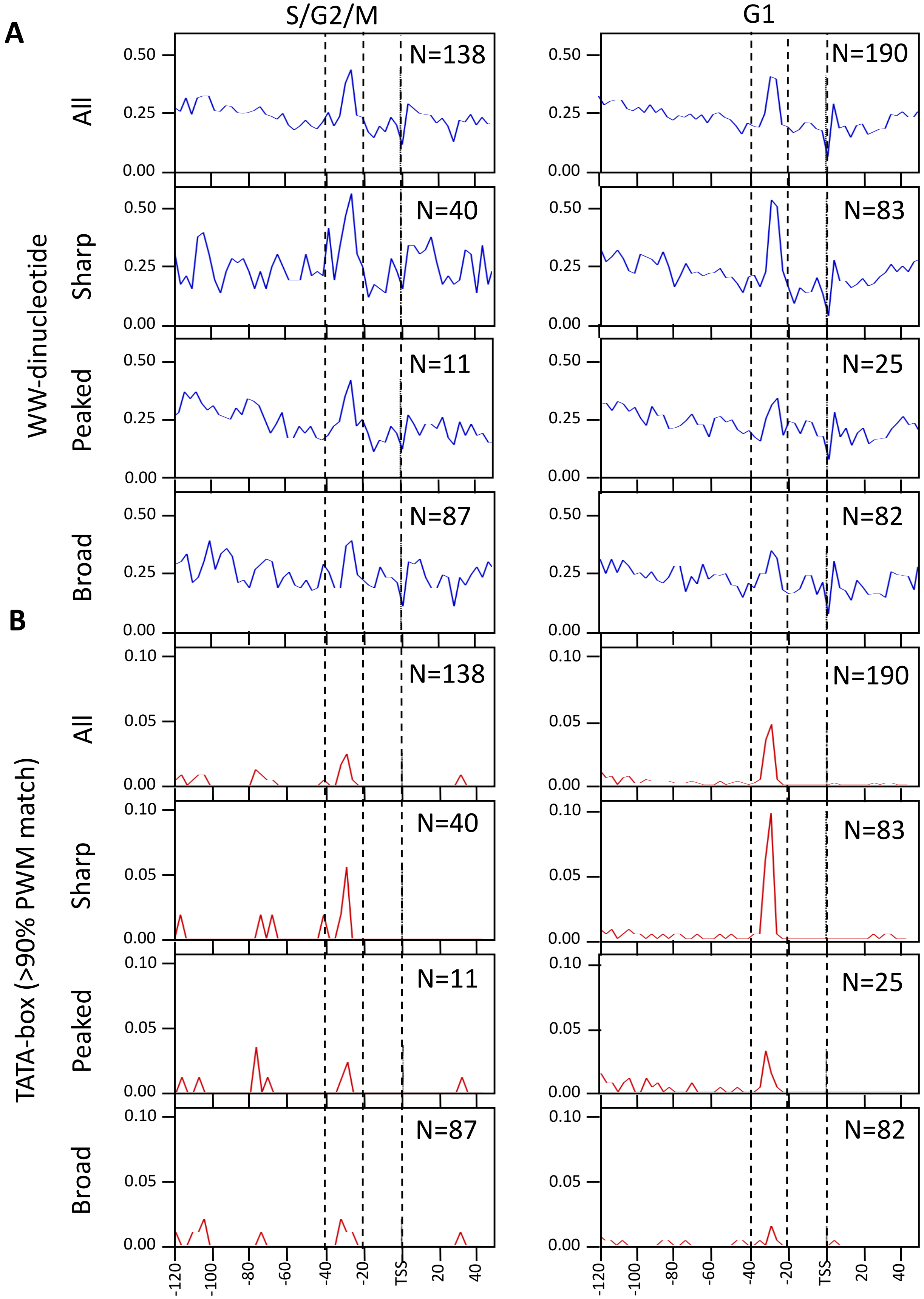

Supplementary figure 5: Analysis of differentially expressed gene promoter features. [A] WW Dinucleotide frequency analysis within the promoter region (120bp downstream and 50bp upstream of the dominant TSS) in genes with different promoter shapes, differentially expressed between G1 and S/G2/M. [B] TATA motif (>90% PWM match) frequency analysis within the promoter region (120bp downstream and 50bp upstream of the dominant TSS) in genes with different promoter shapes, differentially expressed between G1 and S/G2/M.

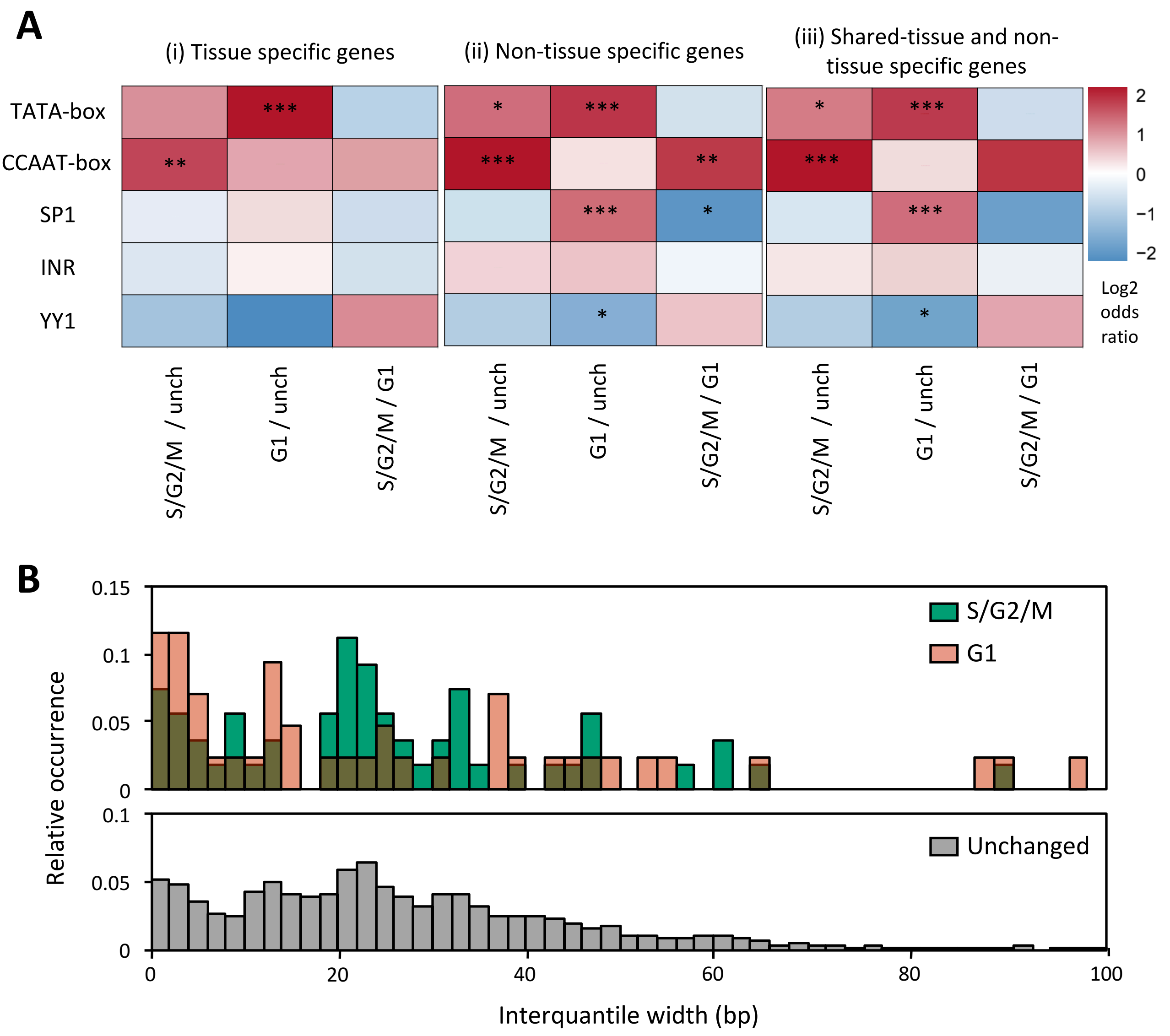

Supplementary figure 6: Differential promoter motif utilisation and TSS distribution in S/G2/M and G1 populations is tissue independent. Identified promoters within the CAGE analysis were assigned tissue specificity as described in Supplementary figure 3. [A] Heatmap visualizing the log2 odds ratio of selected promoter motif occurrence for (i) tissue specific genes, (ii) genes with no tissue specificity and (iii) genes specific to tissues represented in both populations (e.g neural tissues and sensory tissues), or with no tissue specificity – representing a tissue independent comparison set. (*p<0.05, **p<0.01, ***p<0.001, Fisher's exact test). [B] Consensus cluster interquantile width in genes expressed in shared tissues or with no-tissue specificity {A(iii)}, and upregulated in S/G2/M (green) and G1 (red) and with unchanged expression (grey), visualised as a histogram.

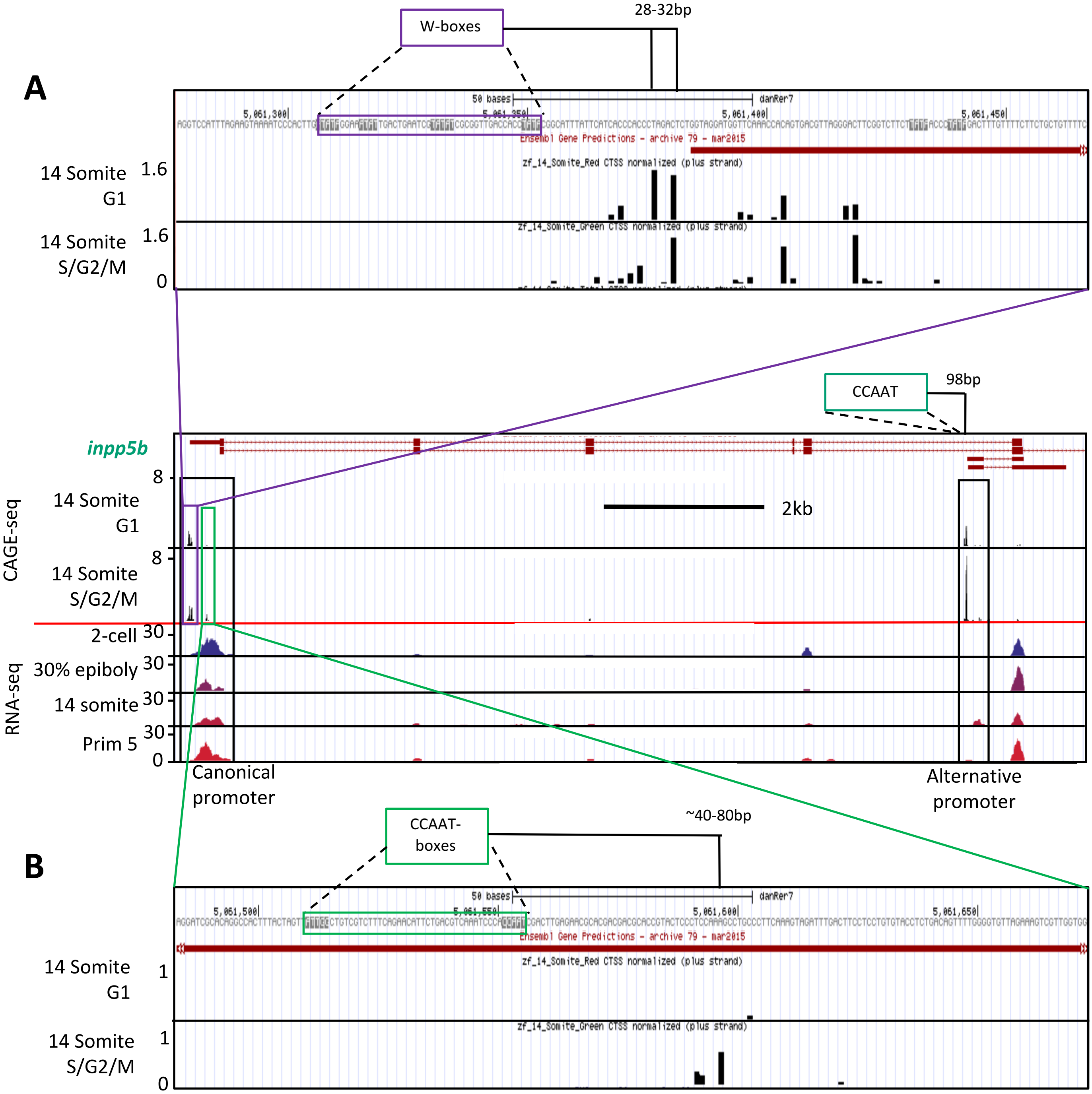

Supplementary Figure 7: Further exploration of the regulation of inpp5b, illustrated in Figure 6D. UCSC genome browser view of the *inositol polyphosphate-5-phosphatase B* (*inpp5b)* promoters, with a focus of the canonical promoter. CAGE-seq tracks show unchanged overall expression from this promoter, but identifies two distinct cell cycle stage specific isoforms, one enriched in G1 proximal to W-box motifs [A] and another S/G2/M specific isoform proximal to two CCAAT-box motifs [B]. RNA-seq tracks, imported from the "Promoterome CAGE and nucleosome positioning" publicly available trackhub (URL: http://trackhub.genereg.net/promoterome/danRer7/index.html) (17, 31) show a bimodal profile of first exon expression, supportive of the dual isoform CAGE profile, particularly at the 14 somite stage.

Supplementary table 1: Proportion of segregated cell displaying 2N-4N DNA content as determined by propidium iodide staining

	2N	2-4N	4N
Total	38.8	16.6	21.6
Red (G1)	80.9	2.3	6.7
Green (S/G2/M)	11	26.6	45.6

Supplementary table 2: Key library and tag cluster statisitics for each sample replicate

Cycle Stage	Sample ID	Library size (in million)	Number of tag clusters	Number of tag clusters in known promoters	Median interquantile width	Mean interquantile width
G1 (Red)	1	10.68	16,057	12,602	15	18.91
	2	7.77	14,339	9,986	6	11.61
	3	10.12	15,829	12,311	15	19.06
S/G2/M (Green)	1	9.74	15,862	12,538	15	18.31
	2	7.79	16,454	12,491	16	19.44
	3	12.80	16,084	12,746	15	18.63
Total	1	7.86	16,227	12,387	15	18.77
	2	4.85	15,051	11,830	14	17.71
	3	7.06	15,080	11,929	15	18.08

Supplementary table 3: Key library and tag cluster statisitics for merged samples

Cycle Stage	Library size (in million)	Number of tag clusters	Number of tag clusters in known promoters	IQ width (median)	IQ width (median)
14_Somite_Red (G1)	30.33	17,133	13,338	15	19.06
14_Somite_Green (S/G2/M)	20.80	16,614	12,949	15	18.94
14_Somite_Total	19.77	17,232	13,212	15	18.91
14_Somite_published_CAGE	5.79	16,531	13,237	14	17.84

Supplementary table 4: TC overlap (%) between each merged sample and previously published CAGE

sampleID	14_Somite_Green(S/G2/M)	14_Somite_Red(G1)	14_Somite_Total	14_Somite_published_CAGE
14_Somite_Green (S/G2/M)	100.00	84.93	81.46	62.31
14_Somite_Red (G1)	87.16	100.00	83.46	63.45
14_Somite_Total	83.19	82.95	100.00	62.47
14_Somite_published_CAGE	68.30	67.64	67.31	100.00

Supplementary table 5: GeneID overlap (%) between each merged sample and previously published CAGE

sampleID	14_Somite_Green(S/G2/M)	14_Somite_Red(G1)	14_Somite_Total	14_Somite_published_CAGE
14_Somite_Green (S/G2/M)	100	94.96249	94.15476	84.68378
14_Somite_Red (G1)	97.13944	100	95.65303	85.95784
14_Somite_Total	95.20582	94.55325	100	85.89368
14_Somite_published_CAGE	92.08612	91.37679	92.37063	100

Supplementary table 6: Intersection between differentially expressed genes between samples and cell cycle periodic genes from Cyclebase

de_group	Number of promoters	lumber of human ortholog	Cell cycle base	Pvalue_permutation
Green (S/G2/M) upregulated	138	92	43	< 1 e-4
non-significant	8406	6158	246	n.s
red (G1) upregulated	190	137	6	n.s

Supplementary table 7: Promoter motif frequency analysis

sampleID	de_group	motif	N	rel_N	count_yes	count_no
14_Somite_Green (S/G2/M)	Green (S/G2/M)_upregulated	SP1	138	0.072463768	10	128
		YY1	138	0.050724638	7	131
		INR	138	0.202898551	28	110
		GC-Box	138	0.072463768	10	128
		CCAAT-Box	138	0.275362319	38	100
		TATA-Box	138	0.086956522	12	126
	non-significant	SP1	8406	0.093028789	782	7624
		YY1	8406	0.094337378	793	7613
		INR	8406	0.180585296	1518	6888
		GC-Box	8406	0.075660243	636	7770
		CCAAT-Box	8406	0.082441113	693	7713
		TATA-Box	8406	0.037116345	312	8094
14_Somite_Red (G1)		SP1	8463	0.09228406	781	7682
		YY1	8463	0.094292804	798	7665
		INR	8463	0.190830675	1615	6848
		GC-Box	8463	0.076923077	651	7812
		CCAAT-Box	8463	0.081176888	687	7776
		TATA-Box	8463	0.038166135	323	8140
	Red (G1)_upregulated	SP1	190	0.168421053	32	158
		YY1	190	0.031578947	6	184
		INR	190	0.242105263	46	144
		GC-Box	190	0.152631579	29	161
		CCAAT-Box	190	0.12631579	24	166
		TATA-Box	190	0.136842105	26	164

Supplementary table 8: A/T pentamer frequency analysis

sampleID	de_group	motif	N	rel_N	count_yes	count_no
14_Somite_Green (S/G2/M)	Green (S/G2/M)_upregulated	AAAAA	138	0.007246377	1	137
		AAAAT	138	0.036231884	5	133
		AAATA	138	0.050724638	7	131
		AAATT	138	0.036231884	5	133
		AATAA	138	0.072463768	10	128
		AATAT	138	0.02173913	3	135
		AATTA	138	0.007246377	1	137
		AATTT	138	0.057971014	8	130
		ATAAA	138	0.108695652	15	123
		ATAAT	138	0.007246377	1	137
		ATATA	138	0.028985507	4	134
		ATATT	138	0.028985507	4	134
		ATTAA	138	0.007246377	0	138
		ATTAT	138	0.007246377	0	138
		ATTTA	138	0.065217391	9	129
		ATTTT	138	0.043478261	6	132
		TAAAA	138	0.108695652	15	123
		TAAAT	138	0.101449275	14	124
		TAATA	138	0.014492754	2	136
		TAATT	138	0.007246377	0	138
		TATAA	138	0.050724638	7	131
		TATAT	138	0.043478261	6	132
		TATTA	138	0.007246377	0	138
		TATTT	138	0.079710145	11	127
		TTAAA	138	0.152173913	21	117
		TTAAT	138	0.007246377	0	138
		TTATA	138	0.02173913	3	135
		TTATT	138	0.007246377	1	137
		TTTAA	138	0.144927536	20	118
		TTTAT	138	0.02173913	3	135
		TTTTA	138	0.014492754	2	136
		TTTTT	138	0.02173913	3	135
		AAAAA	8406	0.033071616	278	8128
		AAAAT	8406	0.022008089	185	8221
		AAATA	8406	0.032476802	273	8133
		AAATT	8406	0.014989293	126	8280
		AATAA	8406	0.023435641	197	8209
		AATAT	8406	0.017130621	144	8262
		AATTA	8406	0.007851535	66	8340
		AATTT	8406	0.016297882	137	8269
		ATAAA	8406	0.053414228	449	7957
		ATAAT	8406	0.011658339	98	8308
		ATATA	8406	0.027837259	234	8172
		ATATT	8406	0.020104687	169	8237
		ATTAA	8406	0.013204854	111	8295
		ATTAT	8406	0.013799667	116	8290
		ATTTA	8406	0.022721865	191	8215
		ATTTT	8406	0.039733524	334	8072
		TAAAA	8406	0.048417797	407	7999
		TAAAT	8406	0.033071616	278	8128
		TAATA	8406	0.012847966	108	8298
		TAATT	8406	0.011777302	99	8307
		TATAA	8406	0.04270759	359	8047
		TATAT	8406	0.027599334	232	8174

	non-significant	TATTA	8406	0.015584107	131	8275
		TATTT	8406	0.039257673	330	8076
		TTAAA	8406	0.040209374	338	8068
		TTAAT	8406	0.013323816	112	8294
		TTATA	8406	0.02022365	170	8236
		TTATT	8406	0.035450868	298	8108
		TTTAA	8406	0.042231739	355	8051
		TTTAT	8406	0.040804187	343	8063
		TTTTA	8406	0.044373067	373	8033
		TTTTT	8406	0.031049251	261	8145
14_Somite_Red (G1)		AAAAA	8463	0.03438497	291	8172
		AAAAT	8463	0.022568829	191	8272
		AAATA	8463	0.03273071	277	8186
		AAATT	8463	0.015242822	129	8334
		AATAA	8463	0.023159636	196	8267
		AATAT	8463	0.017842373	151	8312
		AATTA	8463	0.007916814	67	8396
		AATTT	8463	0.015833629	134	8329
		ATAAA	8463	0.054236086	459	8004
		ATAAT	8463	0.011579818	98	8365
		ATATA	8463	0.029067706	246	8217
		ATATT	8463	0.019496632	165	8298
		ATTAA	8463	0.012997755	110	8353
		ATTAT	8463	0.015006499	127	8336
		ATTTA	8463	0.023159636	196	8267
		ATTTT	8463	0.040056717	339	8124
		TAAAA	8463	0.04785537	405	8058
		TAAAT	8463	0.032021742	271	8192
		TAATA	8463	0.012406948	105	8358
		TAATT	8463	0.011816141	100	8363
		TATAA	8463	0.044192367	374	8089
		TATAT	8463	0.028595061	242	8221
		TATTA	8463	0.015242822	129	8334
		TATTT	8463	0.041592816	352	8111
		TTAAA	8463	0.040647525	344	8119
		TTAAT	8463	0.013115916	111	8352
		TTATA	8463	0.020087439	170	8293
		TTATT	8463	0.036275552	307	8156
		TTTAA	8463	0.04277443	362	8101
		TTTAT	8463	0.041710977	353	8110
		TTTTA	8463	0.044310528	375	8088
		TTTTT	8463	0.03107645	263	8200
	Red (G1)_upregulated	AAAAA	190	0.047368421	9	181
		AAAAT	190	0.021052632	4	186
		AAATA	190	0.042105263	8	182
		AAATT	190	0.010526316	2	188
		AATAA	190	0.021052632	4	186
		AATAT	190	0.031578947	6	184
		AATTA	190	0.005263158	0	190
		AATTT	190	0.010526316	2	188
		ATAAA	190	0.152631579	29	161
		ATAAT	190	0.005263158	1	189
		ATATA	190	0.078947368	15	175
		ATATT	190	0.026315789	5	185
		ATTAA	190	0.010526316	2	188
		ATTAT	190	0.010526316	2	188
		ATTTA	190	0.036842105	7	183
		ATTTT	190	0.036842105	7	183
		TAAAA	190	0.084210526	16	174
		TAAAT	190	0.052631579	10	180
		TAATA	190	0.005263158	1	189
		TAATT	190	0.005263158	1	189
		TATAA	190	0.142105263	27	163
		TATAT	190	0.057894737	11	179
		TATTA	190	0.015789474	3	187
		TATTT	190	0.052631579	10	180
		TTAAA	190	0.073684211	14	176
		TTAAT	190	0.005263158	0	190
		TTATA	190	0.036842105	7	183
		TTATT	190	0.026315789	5	185
		TTTAA	190	0.047368421	9	181
		TTTAT	190	0.021052632	4	186
		TTTTA	190	0.052631579	10	180
		TTTTT	190	0.021052632	4	186
