# Supplementary material for: Embryonic tissue differentiation is characterized by transitions in cell cycle dynamic-associated core promoter regulation
Source: Nucleic Acids Res. 2020 Jul 3;48(15):8374–92. doi: 10.1093/nar/gkaa563 (PMC7470974; doi:10.1093/nar/gkaa563)
Supplement: gkaa563_Supplemental_Files [file gkaa563_supplemental_files.zip › Wragg et al., Supplementary movie legends.docx]

**Supplementary movie 1:** Time-lapse fluorescent imaging of FUCCI embryo development, from high to 19 somite stage, imaged on the lightsheet microscope. Frames were acquired every 15 minutes.

**Supplementary movie 2:** Three dimensional rendering of a FUCCI embryo at the 14 somite stage. Imaged from the dorsal / posterior axis, on the lightsheet microscope.

**Supplementary movie 3:** Three dimensional rendering of a FUCCI embryo at the 14 somite stage. Imaged from the ventral/ anterior axis, on the lightsheet microscope.
